# Supplementary material for: Development and comprehensive evaluation of scarless circularization systems for circular RNA therapeutics
Source: Mol Ther Nucleic Acids. 2025 Jun 9;36(3):102587. doi: 10.1016/j.omtn.2025.102587 (PMC12221454; doi:10.1016/j.omtn.2025.102587)
Supplement: Document S1. Figures S1–S7 [file mmc1.pdf]

OMTN, Volume *36*

Supplemental information

Development and comprehensive evaluation of scarless circularization systems for circular RNA therapeutics

Linfeng Chen, Lianhao Song, Jiaqi Yang, Tong Li, Rong Ju, Caijun Sun, and Zhi Xie

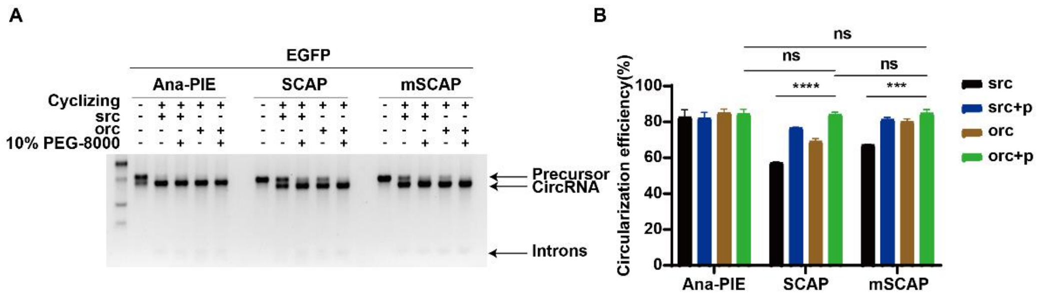

Figure S1. Circularization efficiency across different reaction conditions.

(A) Circularization efficiency of circEGFP produced by the SCAP, mSCAP, and Ana-PIE systems under different reaction conditions.

(B) Quantification of circularization efficiency for circEGFP produced by the SCAP, mSCAP, and Ana-PIE systems under different reaction conditions.

Abbreviations: src, standard reaction condition; src+p, standard reaction condition + 10% PEG-8000; orc, optimized reaction condition; orc+p, optimized reaction condition + 10% PEG-8000 (n = 3; mean ± SEM; ns, not significant; *** $p < 0.001$, **** $p < 0.0001$; unpaired t-test).

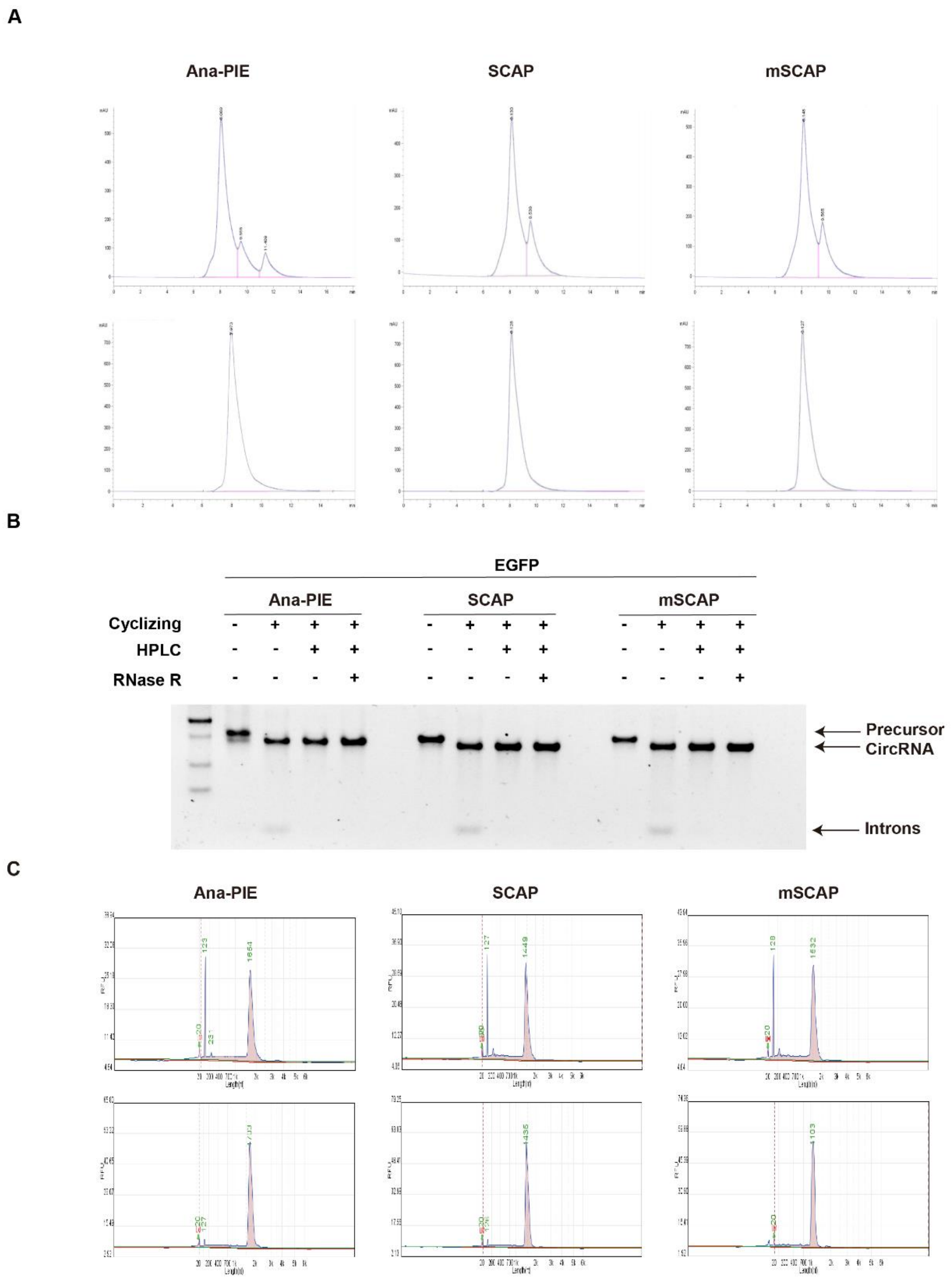

Figure S2. High-purity circRNAs.

(A) HPLC chromatograms of circEGFP before (top) and after (bottom) HPLC purification and RNase R digestion, confirming the high purity of circEGFP.

(B) Agarose gel electropherogram of circEGFP at different stages of purification, showing effective removal of linear precursors and introns.

(C) Capillary electrophoresis analysis of circEGFP before (top) and after (bottom) HPLC purification and RNase R digestion, confirming the purity and homogeneity of the final product.

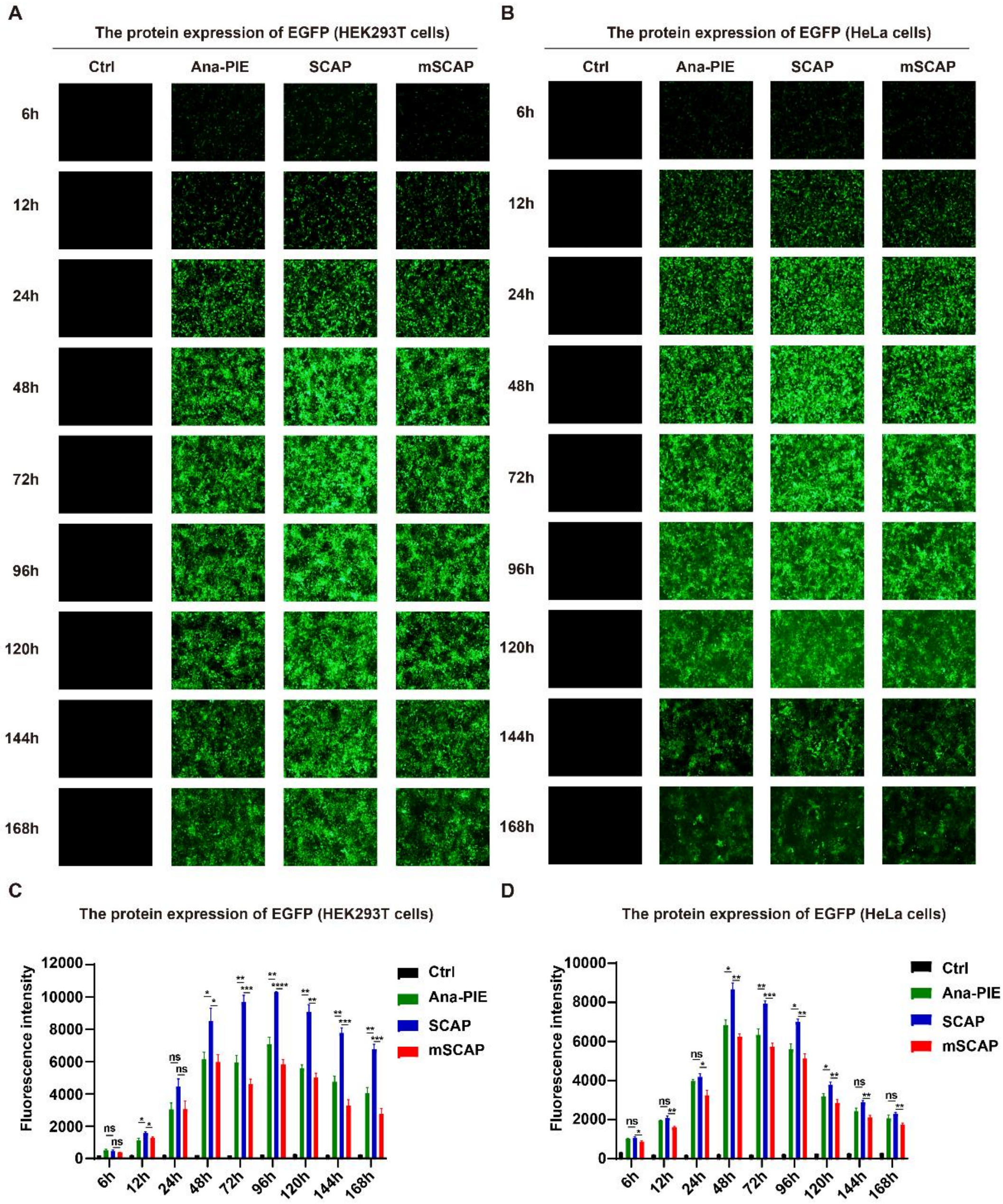

Figure S3. The protein production of EGFP in HEK293T and HeLa cells transfected with EGFP-encoding RNAs.

(A) Fluorescence microscopy images (GFP filter) of HEK293T cells from 6 hours to 168 hours after transfection with EGFP-encoding RNA.

(B) Fluorescence microscopy images (GFP filter) of HeLa cells from 6 hours to 168 hours after transfection with EGFP-encoding RNA.

(C) Fluorescence intensity quantification of HEK293T cells from 6 hours to 168 hours after transfection with EGFP-encoding RNA (n = 3; mean ± SEM).

(D) Fluorescence intensity quantification of HeLa cells from 6 hours to 168 hours after transfection with EGFP-encoding RNA (n = 3; mean ± SEM).

Note: ns, not significant, *p<0.05, ***$p < 0.01$, ****$p < 0.001$, *****$p < 0.0001$; unpaired t-test.

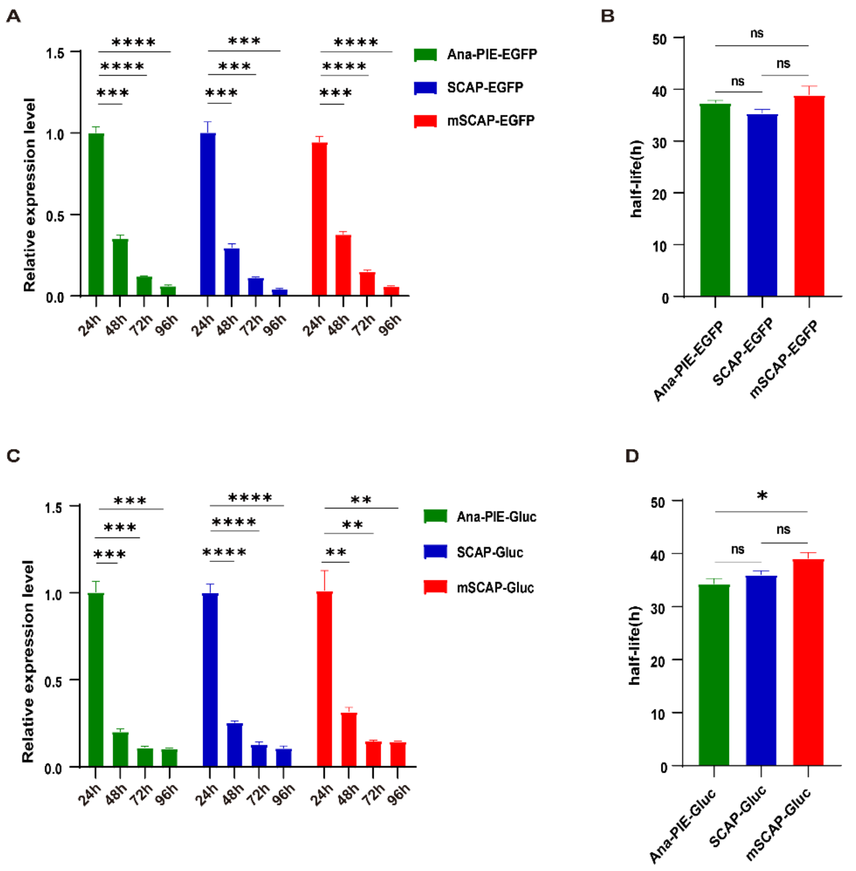

Figure S4. Stability testing of circRNAs in HeLa cells.

(A) The relative expression level of circEGFP, with data normalized to the expression level of the 24-hour time point. (n = 3; mean ± SEM)

(B) The half-life of circEGFP produced by the SCAP, mSCAP, and Ana-PIE systems (n = 3; mean ± SEM).

(C) The relative expression level of circGluc, with data normalized to the expression level of the 24-hour time point (n = 3; mean ± SEM)

(D) The half-life of circGluc produced by the SCAP, mSCAP, and Ana-PIE systems (n = 3; mean ± SEM).

Note: ns, not significant, $*p<0.05$, $**p < 0.01$, $***p < 0.001$, $****p < 0.0001$; unpaired t-test.

A

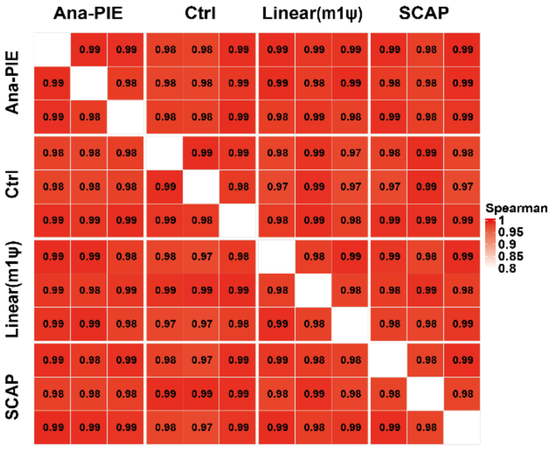

Figure S5. Spearman correlation coefficients for the Ctrl, Ana-PIE, Linear(m1ψ) and SCAP groups.

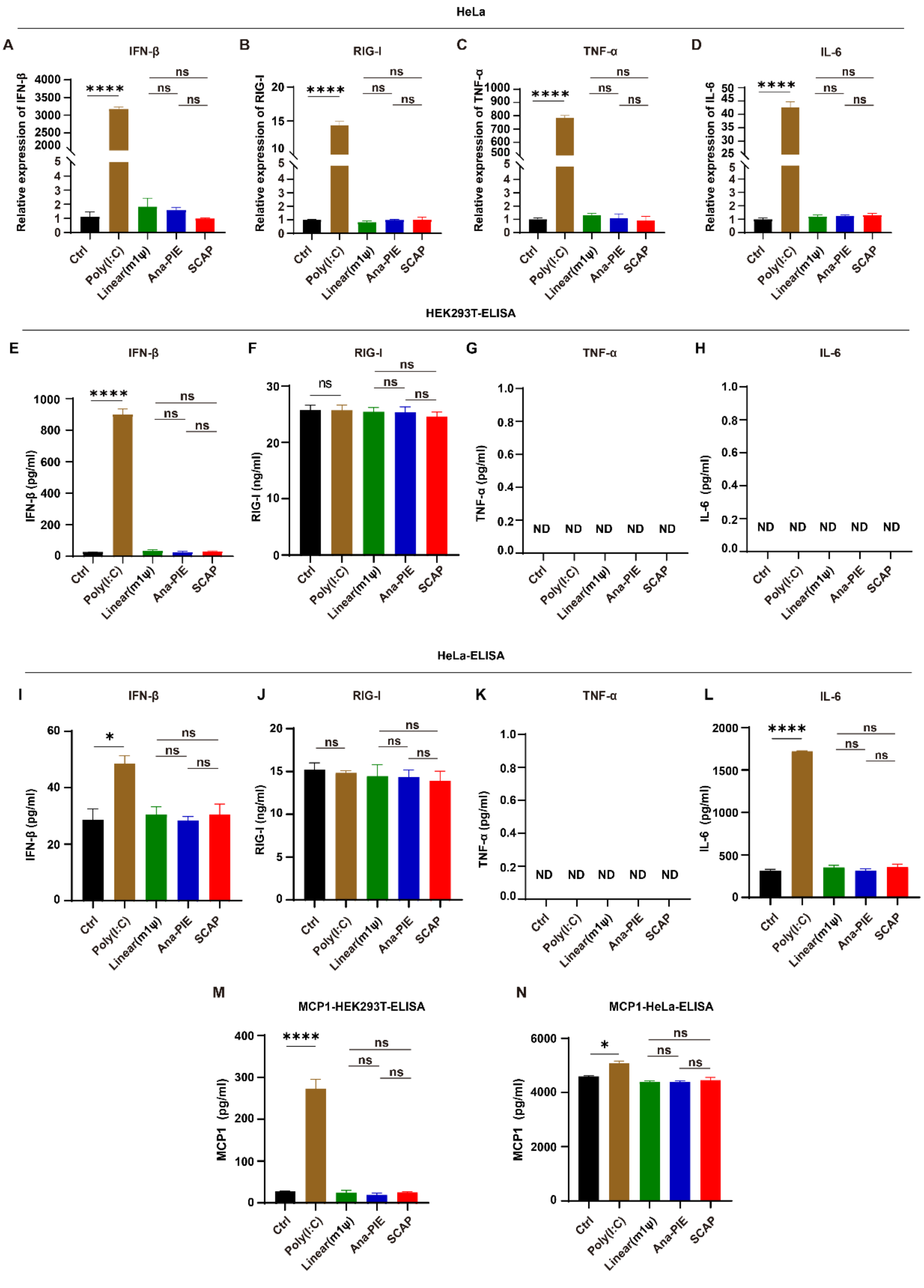

Figure S6. Detection of immune-related factors.

(A-D) mRNA expression of IFN-β, RIG-I, TNF-α, and IL-6 measured by qPCR transfected with Gluc-encoding RNAs at 24 hours in HeLa cells. (n = 3; mean ± SEM).

(E-H, M) Protein expression of IFN-β, RIG-I, TNF-α, IL-6 and MCP1 measured by ELISA transfected with Gluc-encoding RNAs at 48 hours in HEK293T cells ($n = 3$; mean ± SEM).

(I-L, N) Protein expression of IFN-β, RIG-I, TNF-α, IL-6 and MCP1 measured by ELISA transfected with Gluc-encoding RNAs at 48 hours in HeLa cells ($n = 3$; mean ± SEM).

Note: Poly(I:C) was served as a positive control. ns, not significant, * $p < 0.05$, **** $p < 0.0001$; unpaired t-test; ND, not detected.

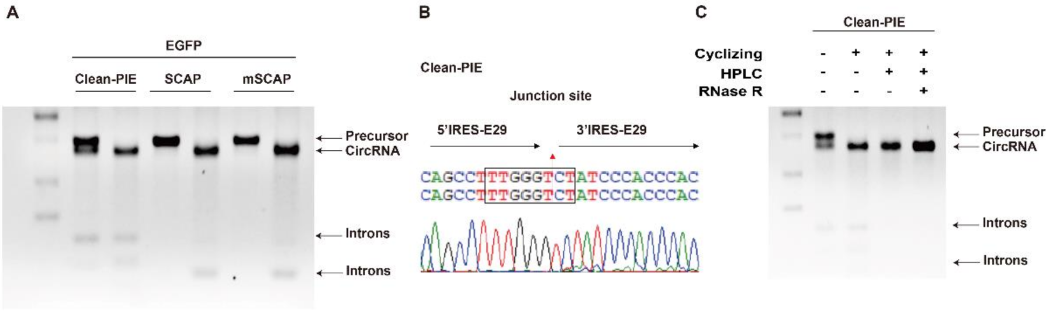

Figure S7. CircRNA synthesis via the Clean-PIE circularization system.

(A) Agarose gel electrophoresis of circEGFP produced by the Clean-PIE, SCAP, and mSCAP systems.

(B) Sanger sequencing of the circEGFP circularization junctions.

(C) Agarose gel electropherogram of circEGFP at different stages of purification, showing effective removal of linear precursors and introns.
