# Supplementary material for: Development and comprehensive evaluation of scarless circularization systems for circular RNA therapeutics
Source: Mol Ther Nucleic Acids. 2025 Jun 9;36(3):102587. doi: 10.1016/j.omtn.2025.102587 (PMC12221454; doi:10.1016/j.omtn.2025.102587)
Supplement: Document S2. Article plus supplemental information [file mmc2.pdf]

Molecular Therapy
Nucleic Acids
Original Article

Development and comprehensive evaluation of scarless circularization systems for circular RNA therapeutics

Linfeng Chen,[1,3] Lianhao Song,[1,3] Jiaqi Yang,[1] Tong Li,[1] Rong Ju,[1] Caijun Sun,[2] and Zhi Xie[1]

[1]State Key Laboratory of Ophthalmology, Zhongshan Ophthalmic Center, Sun Yat-sen University, Guangdong Provincial Key Laboratory of Ophthalmology and Visual Science, Guangzhou 510060, China; [2]School of Public Health (Shenzhen), Sun Yat-sen University, Shenzhen 518107, China

Circular RNAs (circRNAs) are promising candidates for RNA-based therapeutics due to their enhanced stability and sustained protein production compared to linear mRNAs. Traditional circRNA production methods, such as the Anabaena-based permuted intron-exon (Ana-PIE) system, often introduce extraneous sequences, referred to as "scars." However, a comprehensive evaluation of the functional consequences of incorporating or omitting extraneous "scar" sequences during circRNA production is lacking. In this study, we developed two scarless circRNA circularization systems, SCAP and mSCAP, based on Ana-PIE. We systematically compared these systems, along with the scarless Clean-PIE approach, across key performance metrics: circularization efficiency, protein production, stability, and immunogenicity. By quantifying these parameters using multiple reporter genes, we provide a comprehensive evaluation demonstrating that removing scar sequences, particularly with the SCAP system, can enhance protein production while preserving stability and maintaining minimal immunogenicity. Our comprehensive evaluation establishes a framework for the rational design of circRNA therapeutics.

INTRODUCTION

Circular RNAs (circRNAs) are a distinct class of single-stranded, covalently closed RNA molecules formed through a backsplicing process in which a downstream 5′ splice site is ligated to an upstream 3′ splice site.[1–3] This unique closed-loop structure confers several advantages over linear mRNA. Most notably, circRNAs are generally more resistant to exonucleolytic degradation due to the absence of free 5′ or 3′ ends, resulting in enhanced stability and prolonged half-lives within cells.[4–6] As a result, circRNAs can sustain protein production over longer periods, potentially reducing dosing frequency and improving therapeutic outcomes *in vivo*.[7–10]

Various *in vitro* strategies have been devised to generate circRNAs, including chemical ligation, enzymatic ligation, and the use of ribozymes for self-splicing.[11–13] Among these, approaches employing group I intron ribozymes, such as the Anabaena-based permuted intron-exon (Ana-PIE) method, have emerged as particularly versatile and efficient,[7,14] which can circularize relatively large and diverse RNA sequences.[9,15] In the Ana-PIE system, the E1 and E2 sequences (E1E2) are exon sequences derived from the Anabaena intron. Following the permutation of introns and exons, the E1E2 sequences is positioned between the intronic regions, establishing the necessary splice sites for the back-splicing reaction required for circRNA formation.[16] The E1E2 sequences introduce extraneous sequences, often referred to as "scars" that are critical for directing the precise ligation of the RNA ends, thereby ensuring efficient and accurate circularization. While effective, these extraneous elements may potentially introduce some unwanted effects such as increase of immunogenicity.[17,18]

To address these issues and create more native-like circRNAs, researchers have developed new "scarless" circularization methods that avoid inserting these exogenous sequences. For instance, Qiu et al. introduced a Clean-PIE system, which generates circRNAs by circularizing through the protein-coding or internal ribosome entry site (IRES) regions without adding extraneous elements.[19] In a similar vein, Lee et al. and Qi et al. employed end-to-end self-targeting and *cis*-splicing reactions, respectively, to produce scarless circRNAs.[20,21]

Despite these developments, a comprehensive evaluation and consensus on the benefits of "scarless" systems are lacking. Qiu et al., Lee et al., and Qi et al. reported improved protein translation with scarless circRNAs,[19–21] but Hu et al. and Chen et al. found no significant differences.[22,23] Immunogenicity results are similarly mixed: Qiu et al. noted reduced immunogenicity,[19] while Lee et al. and Qi et al. found no difference.[20,21] Stability comparisons are limited, with only Lee et al. reporting increased stability in one

Received 7 January 2025; accepted 5 June 2025;
https://doi.org/10.1016/j.omtn.2025.102587.

[3]These authors contributed equally

Correspondence: Caijun Sun, School of Public Health (Shenzhen), Sun Yat-sen University, Shenzhen 518107, China.
E-mail: suncaijun@mail.sysu.edu.cn
Correspondence: Zhi Xie, State Key Laboratory of Ophthalmology, Zhongshan Ophthalmic Center, Sun Yat-sen University, Guangdong Provincial Key Laboratory of Ophthalmology and Visual Science, Guangzhou 510060, China.
E-mail: xiezhi@gmail.com

© 2025 The Author(s). Published by Elsevier Inc. on behalf of The American Society of Gene and Cell Therapy.
This is an open access article under the CC BY license (http://creativecommons.org/licenses/by/4.0/).

gene.[20] Therefore, a systematic investigation of the scarless and scarred circRNAs, encompassing protein translation, stability, and immunogenicity across multiple constructs and conditions, is required. Factors hindering such studies include the variability in experimental conditions; differences in target genes; and the limited availability of standardized methods for measuring protein production, stability, and immunogenicity. Addressing these challenges would resolve existing discrepancies, clarify the advantages of scarless designs, and guide the rational selection and engineering of circRNA platforms for therapeutic applications.

In this study, we investigate the functional consequences of incorporating or omitting extraneous "scar" sequences during circRNA production. To make a direct comparison of scarred and scarless circularization systems, we engineered two scarless systems based on the established scarred Ana-PIE method. We systematically compared these systems against the original Ana-PIE method and the scarless Clean-PIE approach across key performance metrics: circularization efficiency, protein production, stability, and immunogenicity. By quantifying these parameters using multiple reporter genes, we provide a comprehensive evaluation demonstrating that removing scar sequences using approaches such as the SCAP system, which integrates scar-like elements within the IRES, can enhance protein production while preserving stability. In addition, we found that both the scarred and scarless systems exhibited similarly minimal immunogenicity. Our study provides a framework for evaluating circularization systems and offers important insights for developing circRNA therapies.

RESULTS

Development of "scarless" circular RNA system

The Coxsackievirus B3 (CVB3) IRES is known to support robust and efficient translation of circRNA across multiple cell types.[7] Building on this, we developed a "split-CVB3 Ana-PIE" (SCAP) approach, a scarless circRNA production system that merges the E1E2 sequences into the IRES-CVB3. Specifically, we first identified a segment of the E1E2-like sequences within the IRES-CVB3 that closely resembles the native E1E2 sequences from the Anabaena group I intron by sequences homology search.[24] Based on this segment, we split the IRES-CVB3 into two parts: 5′IRES-CVB3 and 3′IRES-CVB3. These were placed at the respective ends of the linear RNA precursor, and introns along with homology arms were incorporated to promote self-splicing and circularization.[7] This design allows to produce circRNA without introducing extraneous E1E2 sequences (Figure 1A). Alternatively, we could also generate another scarless system by mutating the E2-like sequences within the IRES-CVB3 that precisely match the native E2 sequences, named mutated SCAP (mSCAP) (Figure 1B). Finally, we had two "scarless" systems, SCAP and mSCAP, to compare against the standard Ana-PIE system, which relies on extraneous E1E2 and spacer sequences for circularization.

We first assessed the circularization efficiency of these three methods by testing circRNAs encoding enhanced green fluorescent protein (EGFP).[25] All three approaches demonstrated comparable performance, achieving approximately 80% circularization efficiency (Figures 2A and 2B; materials and methods). These results were corroborated by high-performance liquid chromatography (HPLC) and capillary electrophoresis, which confirmed robust circularization efficiency for all three approaches (Figures S2A and S2C). Furthermore, RNase R digestion, an assay that selectively degrades linear but not circRNA,[26] verified the integrity of circRNAs produced by the three approaches (Figure 2C). After HPLC purification and RNase R digestion, high-purity circRNA was obtained (Figure S2). Sanger sequencing of the circularization junctions confirmed the precise joining of E1E2 in all the cases, confirming accurate and efficient circularization (Figures 2D–2F).

Collectively, we demonstrated that the SCAP and mSCAP systems achieved robust circularization without the inclusion of extraneous spacer and E1E2 sequences and successfully generated scarless circRNAs at high efficiency, comparable to the conventional Ana-PIE method.

Evaluation of protein production

To evaluate how our scarless systems influence translational efficiency of coding sequences, we first generated EGFP-encoding circRNAs (circEGFP) using each of the three circularization systems: SCAP, mSCAP, and Ana-PIE. We then transfected the resulting circEGFP into HEK293T and HeLa cells, respectively, to assess protein production across distinct cellular contexts. After 48 h, circRNAs produced by SCAP led to significantly higher EGFP fluorescence intensity compared to those produced by Ana-PIE and mSCAP (Figures 3A–3C, unpaired t test, $p < 0.05$). Consistent results were observed over a seven-day period, with fluorescence microscopy imaging confirming that SCAP-derived circEGFP consistently maintained higher fluorescence signals relative to the other two methods (Figure S3).

To further validate these findings, we used Gaussia luciferase (Gluc) as an additional reporter,[27] which also offers a sensitive and quantitative readout of protein production. Following transfection of circGluc into HEK293T and HeLa cells, respectively, we measured luciferase activity over four days. Like the EGFP results, SCAP-derived circGluc generated higher peak luciferase activity than both Ana-PIE and mSCAP-derived circGluc (Figures 3D and 3E, unpaired t test, $p < 0.05$ or $p < 0.01$ as indicated). Moreover, SCAP circGluc consistently achieved greater overall luciferase activity throughout the observation period (Figures 3F and 3G, unpaired t test, $p < 0.01$).

To investigate the underlying reasons for these differences in protein yield, we examined the IRES sequence integrity and predicted secondary structures of the circRNAs. Although SCAP and Ana-PIE both incorporate the wild-type IRES-CVB3, Ana-PIE includes exogenous spacer and E1E2 sequences that can adopt independent secondary structures and potentially interfere with IRES function. This added complexity may partially obstruct the initiation of translation and lower protein output. In contrast, SCAP avoids

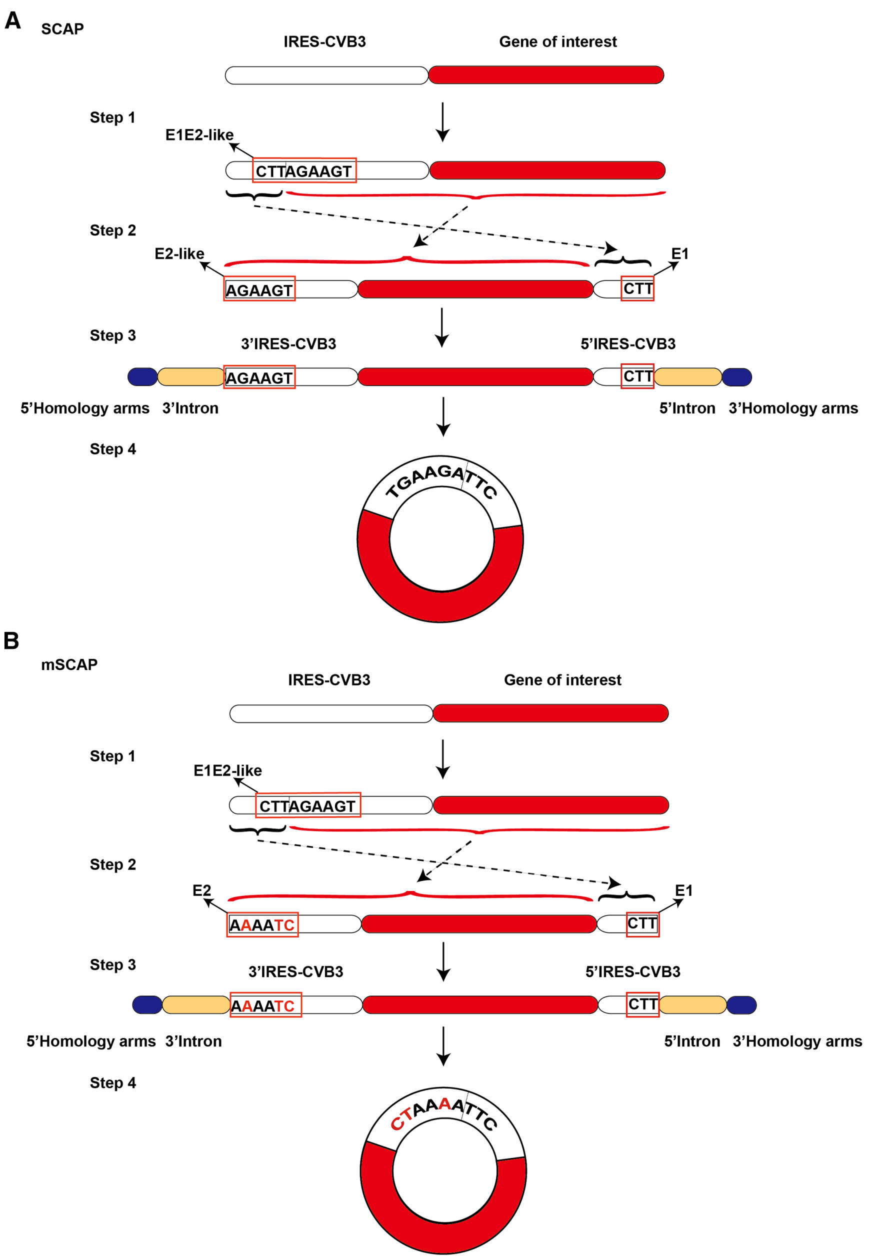

Figure 1. Design of the SCAP and mSCAP

(A) Schematic illustration of the Split-CVB3 Ana-PIE (SCAP) system for scarless circRNA production. Step 1: an endogenous segment within the IRES-CVB3 resembling the Anabaena E1 and E2 sequences (E1E2-like: CTTAGAAGT) is identified. Step 2: the linear RNA precursor design splits the IRES-CVB3 based on these sequences, flanking the gene of interest. Step 3: Anabaena group I introns and homology arms are added to both ends of the linear precursor. Step 4: intron self-splicing and back-splicing reactions mediated by the endogenous E1E2-like sequences result in a covalently closed, scarless circRNA containing the intact IRES-CVB3 and the gene of interest. The junction sequence formed is TGAAGATTC. (B) Schematic illustration of the mutated SCAP (mSCAP) system. Following a similar precursor design as SCAP, the key difference is in step 2: the linear RNA precursor design splits the IRES-CVB3 based on these sequences, flanking the gene of interest. In addition, the endogenous E2-like sequence (AGAAGT) within the 3′ IRES-CVB3 fragment was mutated to precisely match the native Anabaena E2 sequence (AAAATC). Accordingly, the final junction sequence formed is CTAAAATTC in step 4.

introducing extraneous spacer sequences that can form additional secondary structures. Therefore, by preserving the IRES's native sequence and avoiding additional structure, SCAP-derived circRNA likely fosters more efficient recruitment of translation initiation factors, thereby enhancing protein production (Figure 3H).

Meanwhile, mSCAP replaces the IRES's E1E2-like sequences with native Anabaena E1E2 sequences. Although this was intended to facilitate circularization, it appears to have altered critical IRES domains, which refers to a specific region within the IRES sequence that possesses distinct functional or structural characteristics.[28] RNA structure modeling indicated that mSCAP-derived circRNA forms a stem-loop structure at the circularization junction within domain I of the IRES-CVB3 (Figure 3H; materials and methods), an essential region for translation initiation.[29,30] Such sequential and structural disruptions likely weaken the IRES's ability to recruit the translational machinery, resulting in reduced protein production.

In sum, SCAP outperforms Ana-PIE and mSCAP in driving protein production in both EGFP and Gluc reporter systems. This advantage stems from SCAP's ability to maintain a more native, less perturbed IRES environment. The absence of extraneous sequences in SCAP-produced circRNAs may contribute to a more favorable RNA structure for ribosome recruitment, ultimately enhancing translational efficiency.

Evaluation of stability

Assessing the stability of circRNA is crucial for therapeutic applications, as greater stability often correlates with longer *in vivo* half-life and more sustained biological effects.[14] To compare the stability of circRNAs produced by the SCAP, mSCAP, and Ana-PIE systems, we transfected HEK293T and HeLa cells with circEGFP and circGluc constructs and monitored their levels at four time points: 24, 48, 72, and 96 h

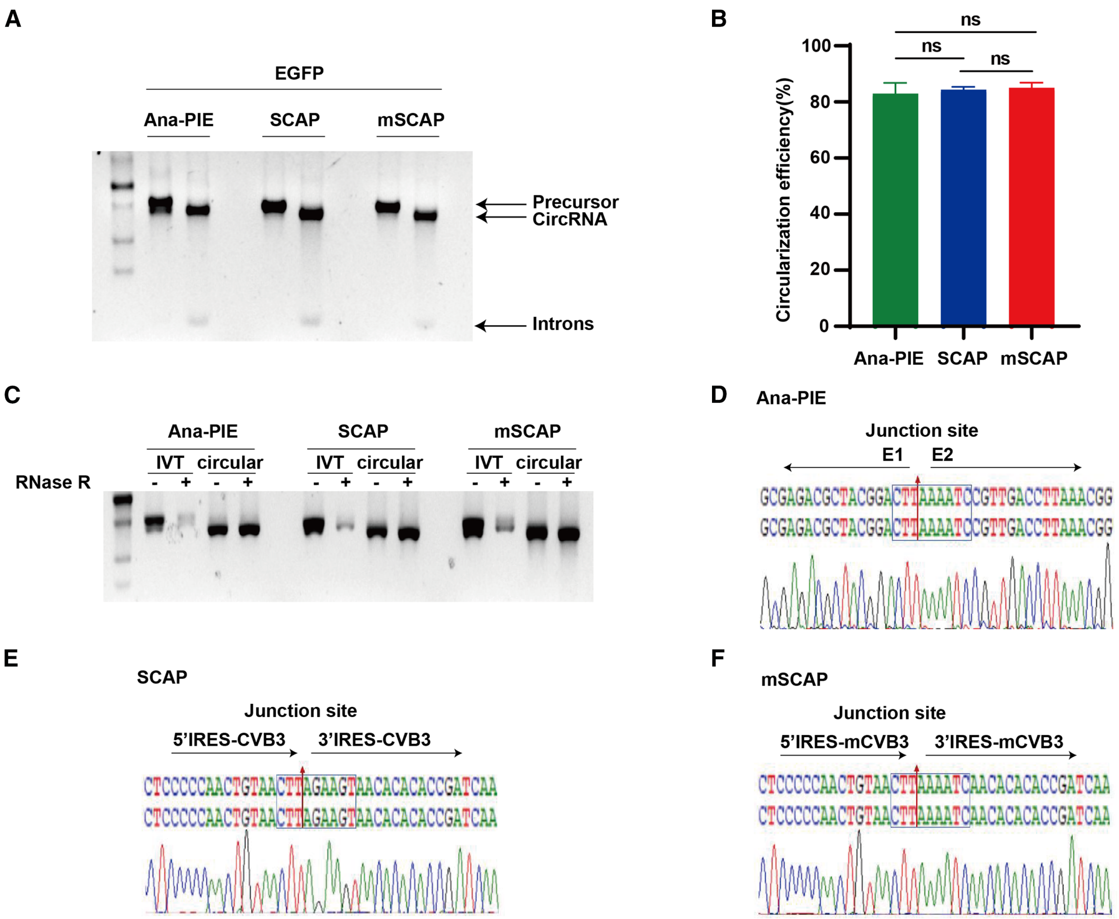

Figure 2. Comparison of circularization efficiency

(A) Agarose gel electrophoresis of circEGFP produced by the SCAP, mSCAP, and Ana-PIE systems. (B) Quantification of circularization efficiency for circEGFP. Results are presented as mean ± SEM (n = 3; ns, not significant; unpaired t test). (C) RNase R digestion of circEGFP and its precursor. (D–F) Sanger sequencing of the circEGFP circularization junctions.

Overall, the relative expression levels of circEGFP and circGluc generated by the three systems were similar (Figures 4A, 4C, S4A, and S4C). Specifically, half-life between SCAP and Ana-PIE-derived circRNAs were similar for either EGFP or Gluc in HEK293T cells (Figures 4B and 4D, unpaired t test; SCAP-EGFP vs. Ana-PIE-EGFP, $p < 0.05$; SCAP-Gluc vs. Ana-PIE-Gluc, $p > 0.05$). These findings were consistent with results obtained in HeLa cells (Figures S4B and S4D, unpaired t test; $p > 0.05$). The mSCAP system generated circRNAs with a slightly longer half-life compared to Ana-PIE (Figures 4B and 4D, unpaired t test, $p < 0.05$ and $p < 0.01$ for EGFP and Gluc, respectively), with similar trends observed in HeLa cells (Figures S4B and S4D, unpaired t test). However, this modest increase in stability was accompanied by reduced protein production levels, potentially limiting mSCAP's utility for applications where high protein output is essential.

Interestingly, the mSCAP circRNA has a less negative minimum free energy (MFE) value (−547.10 kcal/mol) compared to Ana-PIE (−596.90 kcal/mol). The enhanced stability of mSCAP may result from the formation of its stem-loop structure and the mutations introduced within it, which collectively protect circRNA from degradation while preserving overall structural integrity. Previous studies have demonstrated that such stem-loop structures can significantly contribute to RNA stability.[31]

In summary, SCAP-derived circRNAs maintained similar half-lives to those produced by Ana-PIE. While mSCAP offered a slightly stability advantage, its diminished protein production may lessen its appeal for therapeutic use.

Evaluation of immunogenicity

Immunogenicity is a critical factor in the development of RNA-based therapeutics. Previous studies have reported conflicting findings regarding the immunostimulatory effects of extraneous sequences in engineered circRNAs; while some work suggests that these sequences may activate innate immune responses,[17,32] others have

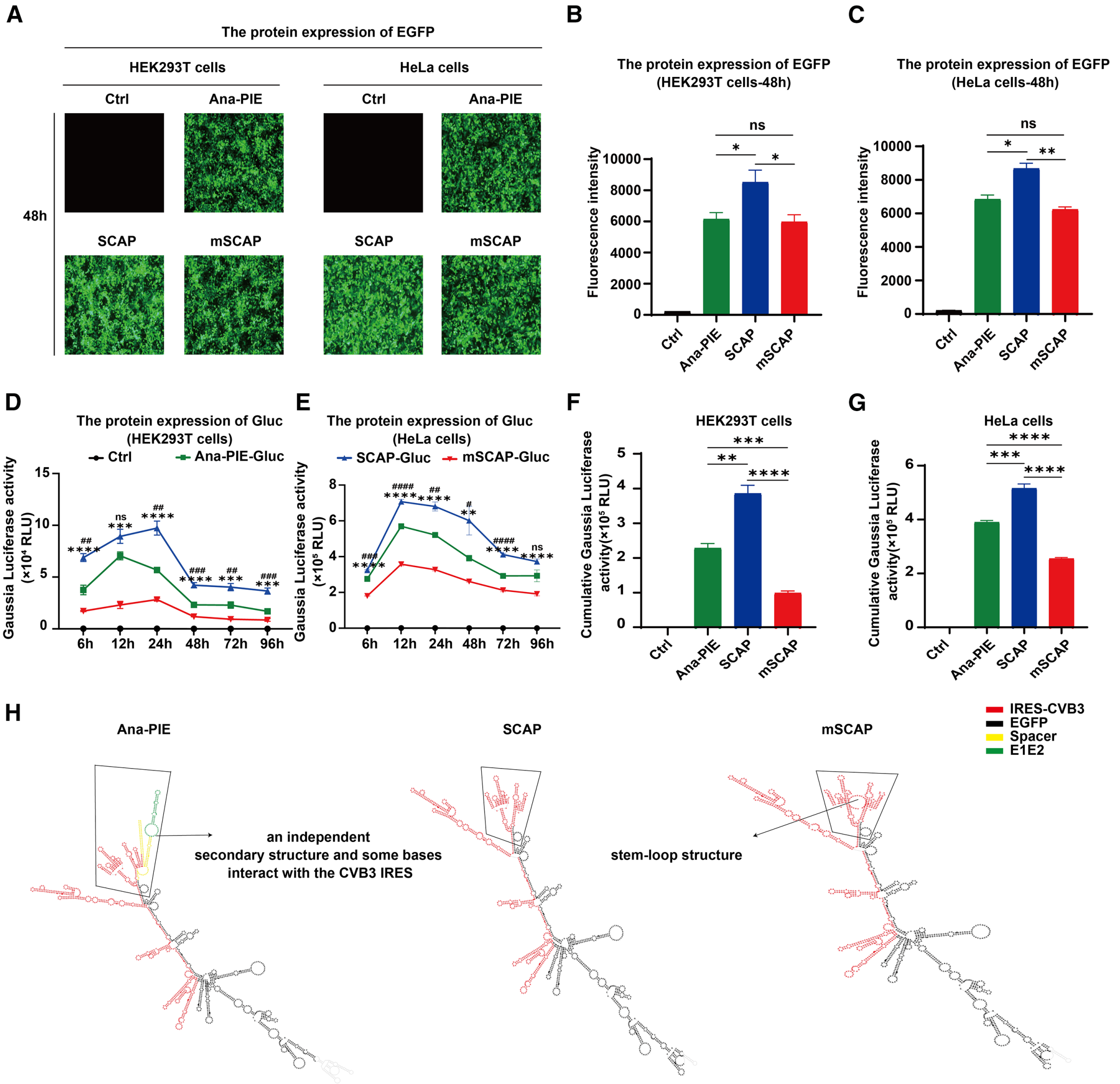

Figure 3. Comparison of protein production

(A) Fluorescence microscopy images were obtained 48 h after transfecting HEK293T and HeLa cells with circRNA. (B and C) Fluorescence intensities of EGFP were measured 48 h post-transfection in HEK293T and HeLa cells ($n = 3$; mean ± SEM). (D and E) A time course of Gaussia luciferase activity was measured in HEK293T and HeLa cells transfected with purified circGluc ($n = 4$; mean ± SEM). (F and G) Cumulative Gaussia luciferase activity over 96 h in HEK293T and HeLa cells ($n = 4$; mean ± SEM). (H) Predicted secondary structures. Note: Ctrl, untreated samples. ns, not significant, #$p < 0.05$, ##$p < 0.01$, ###$p < 0.001$, ####$p < 0.0001$, **$p < 0.01$, ***$p < 0.001$, and ****$p < 0.0001$; unpaired t test.

observed minimal immunogenicity despite their presence.[20,21] However, these previous studies only assessed cellular immunogenicity based on a few immune markers. To clarify the role of exogenous elements, we conducted a comprehensive immunogenicity assessment by performing RNA sequencing (RNA-seq) analysis on SCAP-derived and Ana-PIE-derived circRNAs transfected samples and the untransfected control samples. In addition, we compared these two circRNAs with m1ψ-modified linear RNAs.

Principal-component analysis did not find distinct clustering among the RNA-transfected samples, including linear or circular mRNAs, and the control group (Figure 5A). Moreover, gene expression profiles were highly correlated across all the samples (Figure S4A, Spearman's rho of all the pairwise comparisons >0.97). These analyses indicated no overall transcriptional differences induced by these three mRNA transfections.

Moreover, relative to the untransfected controls, the RNA-transfected samples showed only a few differentially expressed genes, ranging from 2 to 13 (Figure 5B). Gene ontology (GO) analysis revealed no significant GO terms enriched with these differentially expressed genes. Furthermore, gene set enrichment analysis revealed

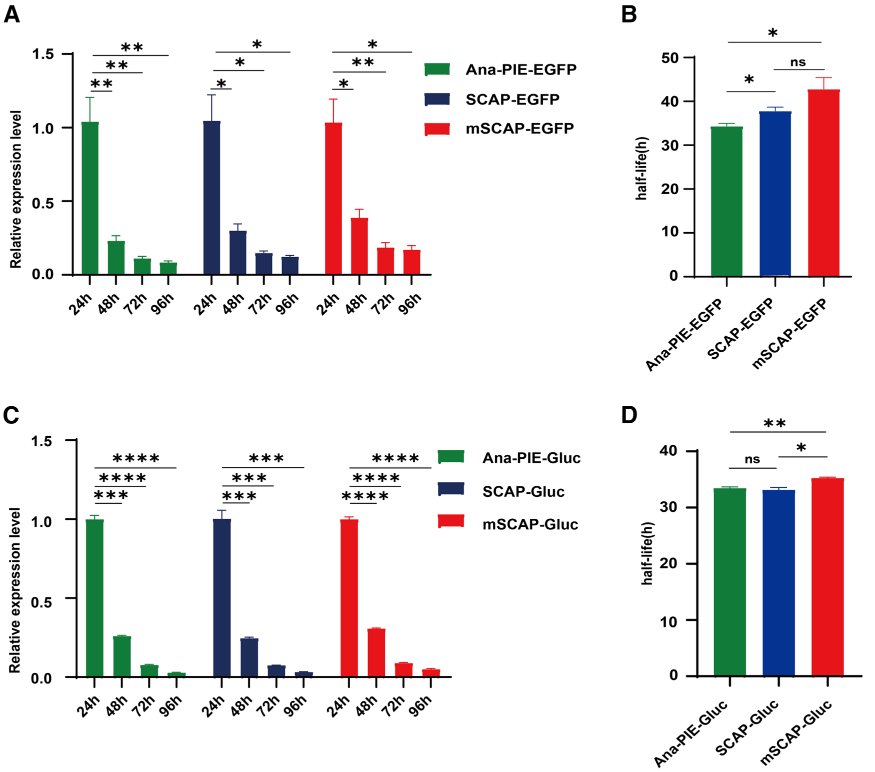

Figure 4. Comparison of stability

(A) The relative expression level of circEGFP, with data normalized to the expression level of the 24-h time point. (n = 4; mean ± SEM). (B) The half-life of circEGFP produced by the SCAP, mSCAP, and Ana-PIE systems (n = 4; mean ± SEM). (C) The relative expression level of circGluc, with data normalized to the expression level of the 24-h time point (n = 4; mean ± SEM). (D) The half-life of circGluc produced by the SCAP, mSCAP, and Ana-PIE systems (n = 4; mean ± SEM). Note: ns, not significant, *p < 0.05, **p < 0.01, ***p < 0.001, and ****p < 0.0001; unpaired t test.

that the top differentially changed pathways were related to innate immune responses, cytokine signaling, and antiviral pathways, although none of them were significantly enriched (Figure 5C, all $p > 0.05$).

We further examined the expression of four known immune markers[17,19,20] using RT-qPCR, including interferon (IFN)-β, RIG-I, interleukin (IL)-6, and tumor necrosis factor alpha (TNF-α). Neither detected notable differences between the SCAP, Ana-PIE, and m1ψ-modified linear RNA groups relative to the untreated controls in HEK293T cells (Figures 5D–5G, unpaired t test, all $p > 0.05$) and HeLa cells (Figures S6A–S6D, unpaired t test, all $p > 0.05$). Additionally, the protein levels of immune-related factors were measured using ELISA, and no significant differences were detected between the experimental groups. Furthermore, none of the groups induced a significant immune response. (Figures S6E–S6N; unpaired t test, all $p > 0.05$). These observations align with some previous reports indicating minimal immunogenicity of engineered circRNAs and modified linear mRNAs.[8,20,21]

In summary, our findings indicated that circRNAs generated by the scarless SCAP system, the traditional Ana-PIE approach, and the tested m1ψ-modified linear RNA all exhibited negligible immunogenicity in the cellular context. The removal of extraneous sequences in the SCAP method did not significantly alter immune responses relative to Ana-PIE-derived circRNA or m1ψ-modified linear RNA.

Comparison with clean-PIE

We finally compared our systems to Clean-PIE, a scarless circularization system that utilizes the td group I intron from bacteriophage T4 (T4td) for circularization and employs the IRES from Echovirus 29 (E29) to drive RNA translation. Using EGFP as a reporter, our experimental results demonstrated that all three systems achieved high circularization efficiency (Figure S7A). Sanger sequencing confirmed that the Clean-PIE system enables precise ligation of the ends of the circRNA precursor (Figure S7B). Furthermore, HPLC purification followed by RNase R digestion effectively removed circularization byproducts (Figure S7C), indicating that the Clean-PIE system produces highly pure and reliable circRNA.

To assess protein production, HEK293T and HeLa cells were transfected with circEGFP constructs generated by each of the three scarless circularization systems. Fluorescence intensity was measured 48 h post-transfection. The results showed that circEGFP produced via the SCAP system exhibited significantly higher protein production compared to the Clean-PIE system. Specifically, in HEK293T cells, the protein production level of circEGFP generated using the SCAP circularization system was 1.8-fold higher than that produced by Clean-PIE. Similarly, in HeLa cells, the protein production level was 1.57-fold higher (Figures 6A–6C; unpaired t test, p = 0.000111 in HEK293T cells, p = 0.000908 in HeLa cells). Regarding stability, the relative RNA levels of circEGFP generated by the three scarless circularization systems were similar (Figure 6D). circEGFP generated via the Clean-PIE system demonstrated a modest increase in stability compared to the SCAP system, with a 1.16-fold longer half-life (Figure 6E, p = 0.036558). The observed differences in protein production and RNA stability between the two systems may be attributed to the distinct circularization system used. Finally, immunogenicity assays revealed that circRNAs produced by the three scarless circularization systems did not elicit significant innate immune responses (Figures 6F–6I, unpaired t test, $p > 0.05$).

In summary, the SCAP circularization system demonstrated higher protein production and a slightly shorter half-life compared to

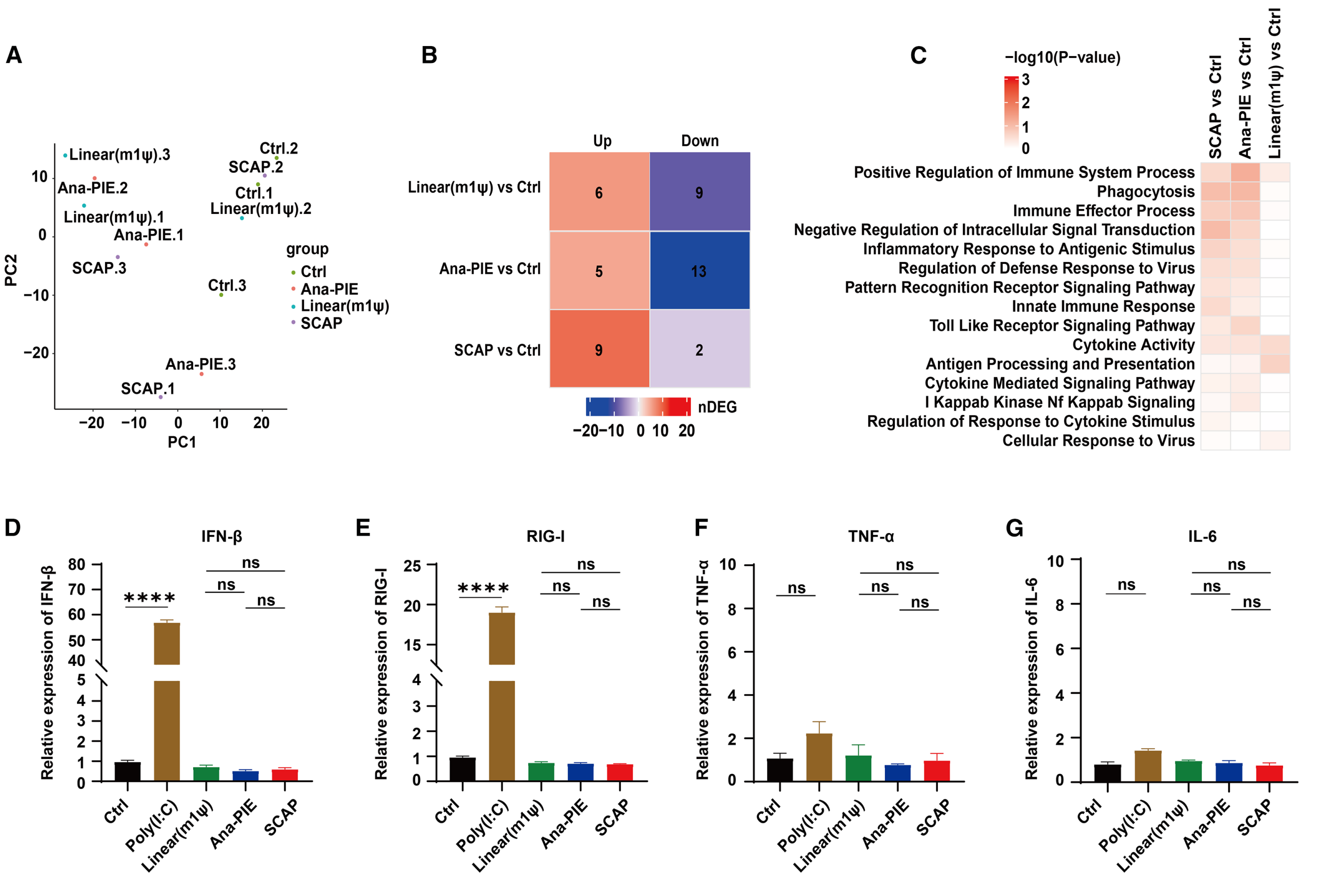

Figure 5. Comparison of immunogenicity

(A) Principal-component analysis of the untreated (Ctrl) and the treatment groups by Gluc mRNAs generated by Linear (m1ψ), Ana-PIE, and SCAP. (B) Differentially expressed gene analysis of the treatment vs. the untreated groups using expression fold change >2 and p value <0.05. (C) Gene set enrichment analysis. (D–G) mRNA expression of IFN-β, RIG-I, TNF-α, and IL-6 measured by qPCR transfected with Gluc-encoding RNAs at 24 h. Poly(I:C) was served as a positive control. ($n = 3$; mean ± SEM; ns, not significant, $^{**}p < 0.01$ and $^{****}p < 0.0001$; unpaired t test).

Clean-PIE, while maintaining comparable circularization efficiency and low immunogenicity. These findings highlight SCAP as a robust and efficient circularization platform, particularly well-suited for applications requiring high-level protein production.

DISCUSSION

In this study, we introduced two scarless circRNA circularization systems, SCAP and mSCAP, which integrate the E1E2 sequences into the IRES region rather than embedding extraneous elements directly into the coding sequences. This design choice distinguishes our approach from previous methods that inserted sequences directly into the open reading frame, which can introduce undesired mutations or disrupt the encoded protein's function.[19] By utilizing the endogenous IRES region, our systems can be readily applied to any coding sequences without compromising protein integrity.

Our evaluation revealed that both scarless circularization systems efficiently generate circRNAs, achieving circularization efficiencies on par with the widely used Ana-PIE method. Notably, this high efficiency was maintained even in SCAP, where the E1E2-like sequences embedded within the IRES-CVB3 differ from the native E1E2 configuration. This suggests that precise replication of the original E1E2 sequences may not be strictly necessary for robust circularization, broadening the design flexibility and applicability of scarless circRNA production strategies.

A key advantage of the SCAP system is its capacity to support enhanced protein production. We observed that SCAP-derived circRNAs yield significantly higher protein production levels than either Ana-PIE- or mSCAP-derived constructs, as demonstrated by EGFP and Gaussia luciferase reporters. This benefit likely arises from preserving the native IRES-CVB3 sequence and its secondary structure, which collectively promote more efficient translation initiation. In contrast, introducing extraneous sequences (Ana-PIE) or altering the IRES (mSCAP) likely disrupts optimal RNA folding and ribosome recruitment.

With respect to stability, SCAP-derived circRNAs proved as durable as those produced by Ana-PIE, displaying comparable

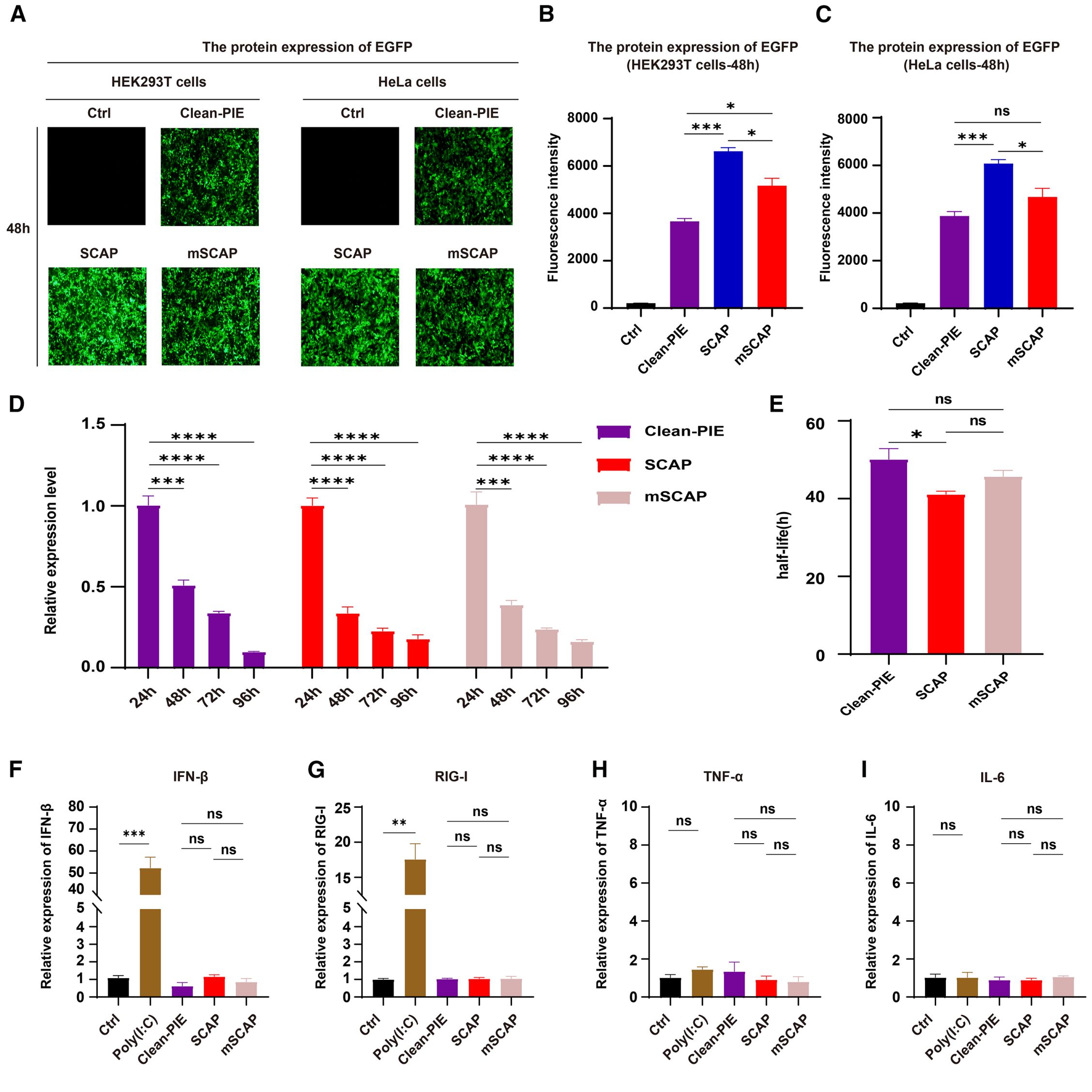

Figure 6. Comparison of Clean-PIE, SCAP and mSCAP

(A) Fluorescence microscopy images were obtained 48 h after transfecting HEK293T and HeLa cells with circRNA. (B and C) Fluorescence intensities of EGFP were measured 48 h post-transfection in HEK293T and HeLa cells ($n = 3$; mean ± SEM). (D) The relative expression level of circEGFP, with data normalized to the expression level of the 24-h time point. ($n = 3$; mean ± SEM). (E) The half-life of circEGFP produced by the Clean-PIE, SCAP, and mSCAP systems ($n = 3$; mean ± SEM). (F–I) mRNA expression of IFN-β, RIG-I, TNF-α, and IL-6 measured by qPCR transfected with Gluc-encoding RNAs at 24 h. Poly(I:C) was served as a positive control. ($n = 3$; mean ± SEM). Note: ns, not significant, *$p < 0.05$, **$p < 0.01$, ***$p < 0.001$, and ****$p < 0.0001$; unpaired t test.

half-lives across multiple days while mSCAP demonstrated a slightly increase in stability. Interestingly, the mSCAP circRNA has a higher MFE value (−547.10 kcal/mol) compared to Ana-PIE (−596.90 kcal/mol). Therefore, the enhanced stability in mSCAP might be attributed to the formation of specific secondary structures, such as the stem-loop at the circularization junction within domain I of the IRES-CVB3. Additionally, the precise mutations introduced in mSCAP could enhance the rigidity or compactness of certain regions, further protecting the circRNA from degradation.

Immunogenicity remains a critical consideration for any RNA therapeutic. Although some previous studies have suggested that removing extraneous sequences can reduce immunogenicity,[17] our analysis indicates no significant difference between scarless and scarred systems in the tested cellular model. Neither the SCAP (scarless) nor the Ana-PIE (scarred) method and the m1ψ-modified linear mRNAs, elicited substantial innate immune responses. This finding suggests that scarless approaches do not inherently confer an immunological advantage under these conditions. Instead, our results imply that both scarless and conventional circRNA designs, as

well as modified linear mRNAs, can maintain negligible immunogenicity, offering flexibility in therapeutic design without increasing the risk of adverse immune reactions.

In addition to SCAP and mSCAP, we further evaluated the Clean-PIE system to broaden our comparative analysis. Consistent with SCAP and mSCAP, Clean-PIE achieved high circularization efficiency and precise junction formation, as well as high final product purity following post-purification analysis. However, despite exhibiting slightly improved RNA stability, circRNAs produced by Clean-PIE showed lower protein production compared to those generated by SCAP. This discrepancy may stem from intrinsic differences in the circularization mechanisms employed by each system. Collectively, these results underscore a critical trade-off between RNA stability and translational efficiency, emphasizing the need for careful selection and optimization of circularization strategies to meet specific therapeutic demands, particularly for applications requiring robust protein expression. Importantly, Clean-PIE also demonstrated minimal immunogenicity, comparable to that observed with SCAP and mSCAP, further supporting the flexibility of these systems for RNA therapeutic development.

While our study provides valuable insights into circRNA design and function, our evaluations were conducted *in vitro* using established cell lines. Although these cell lines are effective for assessing protein production, stability, and immunogenicity, they may not fully recapitulate the complexities of *in vivo* biological systems. In particular, cell lines often differ from primary cells and tissues in RNA metabolism, translation dynamics, and immune responses. Consequently, the behavior of scarred and scarless circularization systems in living organisms may differ from our *in vitro* observations. To bridge this gap, future studies should extend these findings using primary cells and animal models.

In conclusion, our study offers a framework for the rational design of RNA therapeutics. The SCAP system and our comprehensive evaluation provide valuable insights into how balancing circularization efficiency, protein production, stability, and immunogenicity can optimize circRNA production. These findings can inform decisions on where to integrate essential regulatory elements, how to balance stability with protein production, and which modifications to incorporate for the development of mRNA therapies across a broad spectrum of diseases, ultimately accelerating the development of next-generation RNA therapeutics that are both safe and clinically effective.

MATERIALS AND METHODS

Plasmid construction

The Ana-PIE circularization system plasmid was constructed by sequentially inserting the following DNA fragments into the pUC57 vector: a T7 promoter, the Anabaena intron 1 (including homology arm sequences), exon 2, spacer sequence 2, an IRES, the coding sequence, spacer sequence 1, exon 1, and the Anabaena intron 2 (including homology arm sequences). The SCAP and mSCAP plasmids were derived from the Ana-PIE plasmid by removing exon and spacer sequences through PCR-based mutagenesis and homologous recombination. To construct the linear RNA plasmid, a T7 promoter, the 5′ untranslated region (5′ UTR), the coding sequence, the 3′ UTR, and a poly(A) tail were sequentially cloned into the pUC57 vector. In the Clean-PIE plasmid, the E29 and T4td group I intron sequences are derived from the proprietary Clean-PIE technology (patent no. CN114574483B). All plasmid constructs were validated by Sanger sequencing (conducted by Tianyi Huiyuan).

m1ψ-modified linear RNA and circRNA production

For the production of m1ψ-modified linear RNA, the linear RNA plasmid was digested with BspQI and purified using the MinElute Gel Extraction Kit (QIAGEN). Linear RNA was synthesized using the T7 High Yield RNA Synthesis Kit (New England Biolabs) with co-transcriptional capping performed using the m7G(5′)ppp(5′)G RNA Cap Structure Analog (New England Biolabs, S1404) according to the manufacturer's instructions. Following DNase I treatment (New England Biolabs) to remove residual DNA, the RNA was purified using the Monarch RNA Cleanup Kit (New England Biolabs). N1-Me-Pseudo UTP (Yeasen Biotechnology, 10651ES) was used as a substitute for unmodified UTP during transcription.

For the production of circRNA, DNA templates for *in vitro* transcription were linearized by amplification and purified using the MinElute Gel Extraction Kit (QIAGEN). circRNA precursors were synthesized using the T7 High Yield RNA Synthesis Kit (New England Biolabs) following the manufacturer's instructions. After DNase I treatment (New England Biolabs) to remove residual DNA, the circRNA precursors were purified using the Monarch RNA Cleanup Kit (New England Biolabs).

For circRNA production using the Ana-PIE and Clean-PIE systems, purified circRNA precursors were heated at 70°C for 3 min and immediately placed on ice for 2 min. The RNA was then incubated in a reaction mixture containing 1× T4 RNA Ligase Buffer (New England Biolabs) and 2 mM GTP (New England Biolabs) at 55°C for 15 min. Following the reaction, the RNA was purified using a column-based method. The circularization process was conducted in accordance with previously published protocols.[7]

For circRNA production using the SCAP and mSCAP systems, we optimized the reaction conditions by extending the reaction time to 30 min and adjusting the RNA concentration to 200 μg in 600 μL of reaction solution. Additionally, based on previous studies indicating that the inclusion of 10% polyethylene glycol 8000 (PEG-8000) can significantly enhance the *in vitro* self-splicing of group II introns by mimicking cellular conditions,[33,34] we incorporated 10% PEG-8000 into the reaction mixture. Following these optimizations, the circularization efficiency of both the SCAP and mSCAP systems increased substantially, reaching slightly above 80%, which was comparable to the performance of the Ana-PIE system (Figures S1A and S1B).

Purification and verification of circRNA

HPLC was used to separate circRNA based on differences in molecular weight, ensuring effective isolation of the circRNA product.[8,35,36] A 4.6 × 300 mm column (Sepax Technologies, HPLC-26) with a particle size of 5 μm was employed for the separation, with elution carried out at a flow rate of 0.3 mL/min in RNase-free TE buffer (10 mM Tris and 1 mM EDTA [pH 6]). The circRNA was detected at 260 nm and subsequently collected.

Following HPLC purification, the circRNA samples were treated with RNase R (Beyotime) to degrade any remaining linear RNA contaminants, thereby enriching the circRNA product. For RNase R digestion, 1 μg of circRNA was incubated with 1 U of RNase R in 1× RNase R Reaction Buffer at 37°C for 10 min. The reaction mixture was then purified using the Monarch RNA Cleanup Kit (New England Biolabs).

RNA purity and integrity were assessed using the RNA Cartridge Kit (BiOptic) on the Bioptic Qsep 100 Automated Nucleic Acid and Protein Analysis System, which provides high-resolution separation and sensitivity for RNA fragment detection. All procedures followed the manufacturer's instructions.

Reverse transcription PCR and cDNA synthesis

cDNA was synthesized using the HiScript III 1st Strand cDNA Synthesis Kit (Vazyme) via reverse transcription with random primers, using circRNA as the template. Primers flanking the circularization junction were designed to amplify the PCR products for sequencing. After PCR amplification, the products were resolved by agarose gel electrophoresis, excised, purified, and then submitted for sequencing analysis.

Cell culture and transfection

HEK293T and HeLa cells were cultured in DMEM with high glucose, supplemented with 10% fetal bovine serum and 1% dual antibiotic, at 37°C in a 5% CO_2 incubator. Cells were passaged every 2–3 days. For transfection, 293T cells were seeded at a density of 2×10^4 cells per well in 96-well plates and 2×10^5 cells per well in 24-well plates. Once cells reached 70%–90% confluence, RNA was transfected using Lipofectamine MessengerMax (Invitrogen) at 150 ng per well for 96-well plates and 500 ng per well for 24-well plates.

Luciferase activity assay

Carefully aspirate the cell culture medium, and then add 20 μL of 1× cell lysis buffer to each well of a 96-well plate. Incubate for 10 min at room temperature, either by standing or gently shaking. The resulting lysate will be used for subsequent assays. Next, add 50 μL of an 8 μg/mL Renilla reaction solution to each well, mix thoroughly, and incubate in the dark for 1 min. Assays will be performed using an enzyme labeling instrument, with four parallel experiments conducted for each group. Notably, several commercially available kits, such as Vazyme DL101-01, also enable luciferase activity detection through direct cell lysis, and many published studies have adopted such kits for similar assays, underscoring the reliability and reproducibility of this approach.[37–40]

Fluorescence intensity assay

HEK293T (2×10^5) cells were seeded into 24-well plates. Equimolar amounts of RNA were transfected using Lipofectamine MessengerMAX reagent (Invitrogen), following the manufacturer's instructions. Fluorescence intensity of EGFP in transfected cells was measured at designated time points using a Synergy HTX microplate reader (BioTek, Winooski, VT), with an excitation wavelength of 485/20 nm and an emission wavelength of 528/20 nm.

RNA extraction and RT-qPCR

Total RNA was isolated from transfected HEK293T cells using the EZ-press RNA Purification Kit (EZBioscience) and reverse transcribed with the HiScript III RT SuperMix for qPCR (Vazyme). Quantitative PCR was performed on a CFX Connect Real-Time System (Bio-Rad) using iTaq Universal SYBR Green Supermix (Bio-Rad). Relative gene expression was calculated by the $2^{-\Delta\Delta Ct}$ method, with 18S as the reference gene.

RNA secondary structure prediction

RNA secondary structures were predicted using the RNAfold web server (http://rna.tbi.univie.ac.at/cgi-bin/RNAWebSuite/RNAfold.cgi). The circRNA sequences were input into the "Sequence Input" field, with the "assume RNA molecule to be circular" option selected, while keeping the other options at their default settings. RNAfold outputted the predicted secondary structure with MFE.

Determination of RNA half-life

The RNA levels were measured at 24, 48, 72, and 96 h, with data normalized to the 24-h time point. For each sample, we used the following formula to fit the relative RNA levels at different time points:

$$Fi = 100 \times 0.5^{ti/half-life},$$

where Fi is the array of relative RNA levels at multiple time points, and ti represents the time points in hours. The half-life is determined as the fitted decay constant from this analysis.[41]

Enzyme-linked immunosorbent assay

Except for RIG-I, which is a non-secreted protein, all other target proteins are secreted; therefore, RIG-I was quantified using cell lysates. All enzyme-linked immunosorbent assays were performed strictly in accordance with the manufacturers' instructions provided with each kit. Due to differences in kit availability and supplier-imposed constraints for certain assay kits, Enzyme-linked immunosorbent assay (ELISA) for IFN-β, TNF-α, and IL-6 were conducted using kits from HUABIO; the RIG-I assay was carried out using a kit from Afbio; and the MCP-1 assay was performed using a kit from Elabscience.

Library preparation for RNA sequencing

A total of 2 μg of RNA per sample was used as input material for RNA sample preparation. Sequencing libraries were generated using the NEBNext Ultra RNA Library Prep Kit for Illumina (no. E7530L, NEB, USA), following the manufacturer's instructions, with index adapters incorporated to assign sequences to individual samples.

Briefly, mRNA was purified from total RNA using poly-T oligo-attached magnetic beads. RNA fragmentation was achieved with divalent cations under elevated temperature in NEBNext First Strand Synthesis Reaction Buffer (5×). First-strand cDNA synthesis was performed using random hexamer primers and RNase H. Subsequently, second-strand cDNA synthesis was carried out with buffer, dNTPs, DNA polymerase I, and RNase H. The library fragments were purified using QiaQuick PCR kits and eluted in EB buffer. Following purification, terminal repair, A-tailing, and adapter ligation were performed. The desired fragments were selected, followed by PCR amplification to complete the library preparation process.

Library clustering and sequencing

Index-coded samples were clustered using the cBot cluster generation system with the HiSeq PE Cluster Kit v4-cBot-HS (Illumina), following the manufacturer's instructions. After cluster generation, the libraries were sequenced on an Illumina platform, generating 150 bp paired-end reads.

Bioinformatical analysis

The raw RNA-seq data were preprocessed and subjected to quality control using fastp.[42] The human rRNA reference sequence was obtained from NCBI. Bowtie2 was used to construct the reference index and perform local alignment, effectively filtering out reads that mapped to the rRNA sequences.[43] The human genome reference and gene annotation files were sourced from Ensembl. STAR was employed to build the reference index and perform the alignment, followed by RSEM for quantification of gene expression from the resulting alignment data.[44,45] Gene expression normalization and identification of differentially expressed genes were performed using DESeq2.[46] GO enrichment analysis was performed using fGSEA.[47]

Statistical analysis

Data are presented as mean ± standard error of the mean (SEM) from at least three independent experiments. Statistical significance was determined using unpaired t test, with *p*-values <0.05 considered statistically significant. All statistical analyses were performed using GraphPad Prism 8 software (GraphPad Software Inc.).

DATA AVAILABILITY

The sequencing data have been deposited in the Gene Expression Omnibus (GEO) under accession no. GSE284757 and will be made publicly available upon the acceptance of this manuscript.

ACKNOWLEDGMENTS

This work was supported by the National Natural Science Foundation of China (32470705, Z.X.), the Key Area Research and Development Program of Guangdong Province (2023B1111020006, Z.X.), and the Science and Technology Program of Guangzhou, China (2025A03J3990, Z.X.). We gratefully acknowledge the long-term support of the Zhongshan Ophthalmic Center, Sun Yat-sen University.

AUTHOR CONTRIBUTIONS

Z.X. conceived and supervised the study. C.S. co-supervised the study. L.C. conducted experiments. J.Y. and L.C. prepared RNA. L.S. computationally designed SCAP/mSCAP and conducted sequencing data analysis. T.L. and R.J. helped interpret experiments. L.C. and Z. X. wrote the manuscript. All authors read and approved the manuscript.

DECLARATION OF INTERESTS

The authors declare no competing interests.

SUPPLEMENTAL INFORMATION

Supplemental information can be found online at https://doi.org/10.1016/j.omtn.2025.102587.

REFERENCES

1. Chen, L.L. (2016). The biogenesis and emerging roles of circular RNAs. Nat. Rev. Mol. Cell Biol. *17*, 205–211.
2. Kristensen, L.S., Andersen, M.S., Stagsted, L.V.W., Ebbesen, K.K., Hansen, T.B., and Kjems, J. (2019). The biogenesis, biology and characterization of circular RNAs. Nat. Rev. Genet. *20*, 675–691.
3. Zhang, X.O., Wang, H.B., Zhang, Y., Lu, X., Chen, L.L., and Yang, L. (2014). Complementary sequence-mediated exon circularization. Cell *159*, 134–147.
4. Jeck, W.R., and Sharpless, N.E. (2014). Detecting and characterizing circular RNAs. Nat. Biotechnol. *32*, 453–461.
5. Chen, L.L., and Yang, L. (2015). Regulation of circRNA biogenesis. RNA Biol. *12*, 381–388.
6. Enuka, Y., Lauriola, M., Feldman, M.E., Sas-Chen, A., Ulitsky, I., and Yarden, Y. (2016). Circular RNAs are long-lived and display only minimal early alterations in response to a growth factor. Nucleic Acids Res. *44*, 1370–1383.
7. Wesselhoeft, R.A., Kowalski, P.S., and Anderson, D.G. (2018). Engineering circular RNA for potent and stable translation in eukaryotic cells. Nat. Commun. *9*, 2629.
8. Wesselhoeft, R.A., Kowalski, P.S., Parker-Hale, F.C., Huang, Y., Bisaria, N., and Anderson, D.G. (2019). RNA Circularization Diminishes Immunogenicity and Can Extend Translation Duration In Vivo. Mol. Cell *74*, 508–520.e4.
9. Qu, L., Yi, Z., Shen, Y., Lin, L., Chen, F., Xu, Y., Wu, Z., Tang, H., Zhang, X., Tian, F., et al. (2022). Circular RNA vaccines against SARS-CoV-2 and emerging variants. Cell *185*, 1728–1744.e16.
10. Jiang, W., Xiao, D., Wu, C., Yang, J., Peng, X., Chen, L., Zhang, J., Zha, G., Li, W., Ju, R., et al. (2024). Circular RNA-based therapy provides sustained and robust neuroprotection for retinal ganglion cells. Mol. Ther. Nucleic Acids *35*, 102258.
11. Petkovic, S., and Müller, S. (2015). RNA circularization strategies in vivo and in vitro. Nucleic Acids Res. *43*, 2454–2465.
12. Beaudry, D., and Perreault, J.P. (1995). An efficient strategy for the synthesis of circular RNA molecules. Nucleic Acids Res. *23*, 3064–3066.
13. Micura, R. (1999). Cyclic Oligoribonucleotides (RNA) by Solid-Phase Synthesis. Chem. Eur J. *5*, 2077–2082.
14. Li, H., Peng, K., Yang, K., Ma, W., Qi, S., Yu, X., He, J., Lin, X., and Yu, G. (2022). Circular RNA cancer vaccines drive immunity in hard-to-treat malignancies. Theranostics *12*, 6422–6436.
15. Chen, X., and Lu, Y. (2021). Circular RNA: Biosynthesis in vitro. Front. Bioeng. Biotechnol. *9*, 787881.
16. Puttaraju, M., and Been, M.D. (1992). Group I permuted intron-exon (PIE) sequences self-splice to produce circular exons. Nucleic Acids Res. *20*, 5357–5364.
17. Liu, C.X., Guo, S.K., Nan, F., Xu, Y.F., Yang, L., and Chen, L.L. (2022). RNA circles with minimized immunogenicity as potent PKR inhibitors. Mol. Cell *82*, 420–434.e6.
18. Wang, L., Dong, C., Zhang, W., Ma, X., Rou, W., Yang, K., Cui, T., Qi, S., Yang, L., Xie, J., et al. (2024). Developing an enhanced chimeric permuted intron-exon system for circular RNA therapeutics. Theranostics *14*, 5869–5882.
19. Qiu, Z., Hou, Q., Zhao, Y., Zhu, J., Zhai, M., Li, D., Li, Y., Liu, C., Li, N., Cao, Y., et al. (2022). Clean-PIE: a novel strategy for efficiently constructing precise circRNA with thoroughly minimized immunogenicity to direct potent and durable protein expression. Preprint at bioRxiv. https://doi.org/10.1101/2022.06.20.496777.
20. Lee, K.H., Kim, S., Song, J., Han, S.R., Kim, J.H., and Lee, S.-W. (2023). Efficient circular RNA engineering by end-to-end self-targeting and splicing reaction using Tetrahymena group I intron ribozyme. Mol. Ther. Nucleic Acids *33*, 587–598.

21. Qi, S., Wang, H., Liu, G., Qin, Q., Gao, P., and Ying, B. (2024). Efficient circularization of protein-encoding RNAs via a novel cis-splicing system. Nucleic Acids Res. *52*, 10400–10415.

22. Hu, Q., Zhao, H., Zhou, K., Hua, X., and Zhang, X. (2024). Scarless circular mRNA-based CAR-T cell therapy elicits superior anti-tumor efficacy. Preprint at bioRxiv. https://doi.org/10.1101/2024.08.05.606578.

23. Chen, C., Wei, H.-H., Zhang, K., Li, Z., Wei, T., Tang, C., Yang, Y., and Wang, Z.J.b. (2022). A flexible, efficient, and scalable platform to produce circular RNAs as new therapeutics. Preprint at bioRxiv. https://doi.org/10.1101/2022.05.31.494115.

24. Rausch, J.W., Heinz, W.F., Payea, M.J., Sherpa, C., Gorospe, M., and Le Grice, S.F.J. (2021). Characterizing and circumventing sequence restrictions for synthesis of circular RNA in vitro. Nucleic Acids Res. *49*, e35.

25. Gurunathan, S., Woong Han, J., Kim, E., Kwon, D.N., Park, J.K., and Kim, J.H. (2014). Enhanced green fluorescent protein-mediated synthesis of biocompatible graphene. J. Nanobiotechnology *12*, 41.

26. Suzuki, H., Zuo, Y., Wang, J., Zhang, M.Q., Malhotra, A., and Mayeda, A. (2006). Characterization of RNase R-digested cellular RNA source that consists of lariat and circular RNAs from pre-mRNA splicing. Nucleic Acids Res. *34*, e63.

27. Wurdinger, T., Badr, C., Pike, L., De Kleine, R., Weissleder, R., Breakefield, X.O., and Tannous, B.A. (2008). A secreted luciferase for ex vivo monitoring of in vivo processes. Nat. Methods *5*, 171–173.

28. Bailey, J.M., and Tapprich, W.E. (2007). Structure of the 5' nontranslated region of the coxsackievirus b3 genome: Chemical modification and comparative sequence analysis. J. Virol. *81*, 650–668.

29. Murray, K.E., Steil, B.P., Roberts, A.W., and Barton, D.J. (2004). Replication of poliovirus RNA with complete internal ribosome entry site deletions. J. Virol. *78*, 1393–1402.

30. Chen, R., Wang, S.K., Belk, J.A., Amaya, L., Li, Z., Cardenas, A., Abe, B.T., Chen, C.K., Wender, P.A., and Chang, H.Y. (2023). Engineering circular RNA for enhanced protein production. Nat. Biotechnol. *41*, 262–272.

31. Emory, S.A., Bouvet, P., and Belasco, J.G. (1992). A 5'-terminal stem-loop structure can stabilize mRNA in Escherichia coli. Genes Dev. *6*, 135–148.

32. Wilusz, J.E. (2019). Circle the Wagons: Circular RNAs Control Innate Immunity. Cell *177*, 797–799.

33. Lambert, D., and Draper, D.E. (2007). Effects of Osmolytes on RNA Secondary and Tertiary Structure Stabilities and RNA-Mg2+ Interactions. J. Mol. Biol. *370*, 993–1005.

34. Liu, T., and Pyle, A.M. (2021). Discovery of highly reactive self-splicing group II introns within the mitochondrial genomes of human pathogenic fungi. Nucleic Acids Res. *49*, 12422–12432.

35. Rabinovich, P. Synthetic Messenger RNA and Cell Metabolism Modulation: Methods and Protocols [M]. 2013.

36. Carmona, E. M. (2019). Circular RNA: Design Criteria for Optimal Therapeutical Utility, F, [C].

37. Chen, S., Wu, J.L., Liang, Y., Tang, Y.G., Song, H.X., Wu, L.L., Xing, Y.F., Yan, N., Li, Y.T., Wang, Z.Y., et al. (2021). Arsenic Trioxide Rescues Structural p53 Mutations through a Cryptic Allosteric Site. Cancer Cell *39*, 225–239.e8.

38. Zhao, Y., Cao, X., Zhong, W., Zhou, S., Li, Z., An, H., Liu, X., Wu, R., Bohora, S., Wu, Y., et al. (2022). A viral protein orchestrates rice ethylene signaling to coordinate viral infection and insect vector-mediated transmission. Mol. Plant *15*, 689–705.

39. Lin, J.W., Tang, C., Wei, H.C., Du, B., Chen, C., Wang, M., Zhou, Y., Yu, M.X., Cheng, L., Kuivanen, S., et al. (2021). Genomic monitoring of SARS-CoV-2 uncovers an Nsp1 deletion variant that modulates type I interferon response. Cell Host Microbe *29*, 489–502.e8.

40. Liu, Y., Shi, M., He, X., Cao, Y., Liu, P., Li, F., Zou, S., Wen, C., Zhan, Q., Xu, Z., et al. (2022). LncRNA-PACERR induces pro-tumour macrophages via interacting with miR-671-3p and m6A-reader IGF2BP2 in pancreatic ductal adenocarcinoma. J. Hematol. Oncol. *15*, 52.

41. Zhang, H., Zhang, L., Lin, A., Xu, C., Li, Z., Liu, K., Liu, B., Ma, X., Zhao, F., Jiang, H., et al. (2023). Algorithm for optimized mRNA design improves stability and immunogenicity. Nature *621*, 396–403.

42. Chen, S. (2023). Ultrafast one-pass FASTQ data preprocessing, quality control, and deduplication using fastp. Imeta *2*, e107.

43. Langmead, B., and Salzberg, S.L. (2012). Fast gapped-read alignment with Bowtie 2. Nat. Methods *9*, 357–359.

44. Dobin, A., Davis, C.A., Schlesinger, F., Drenkow, J., Zaleski, C., Jha, S., Batut, P., Chaisson, M., and Gingeras, T.R. (2013). STAR: ultrafast universal RNA-seq aligner. Bioinformatics *29*, 15–21.

45. Li, B., and Dewey, C.N. (2011). RSEM: accurate transcript quantification from RNA-Seq data with or without a reference genome. BMC Bioinf. *12*, 323.

46. Love, M.I., Huber, W., and Anders, S. (2014). Moderated estimation of fold change and dispersion for RNA-seq data with DESeq2. Genome Biol. *15*, 550.

47. Subramanian, A., Tamayo, P., Mootha, V.K., Mukherjee, S., Ebert, B.L., Gillette, M.A., Paulovich, A., Pomeroy, S.L., Golub, T.R., Lander, E.S., and Mesirov, J.P. (2005). Gene set enrichment analysis: a knowledge-based approach for interpreting genome-wide expression profiles. Proc. Natl. Acad. Sci. USA *102*, 15545–15550.

OMTN, Volume *36*

Supplemental information

Development and comprehensive evaluation of scarless circularization systems for circular RNA therapeutics

Linfeng Chen, Lianhao Song, Jiaqi Yang, Tong Li, Rong Ju, Caijun Sun, and Zhi Xie

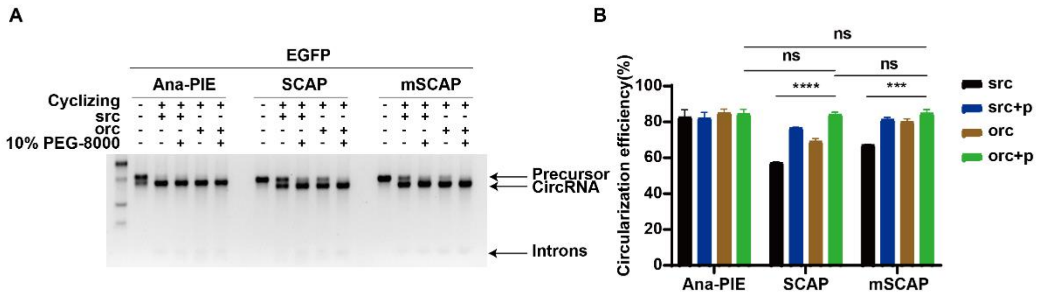

Figure S1. Circularization efficiency across different reaction conditions.

(A) Circularization efficiency of circEGFP produced by the SCAP, mSCAP, and Ana-PIE systems under different reaction conditions.

(B) Quantification of circularization efficiency for circEGFP produced by the SCAP, mSCAP, and Ana-PIE systems under different reaction conditions.

Abbreviations: src, standard reaction condition; src+p, standard reaction condition + 10% PEG-8000; orc, optimized reaction condition; orc+p, optimized reaction condition + 10% PEG-8000 (n = 3; mean ± SEM; ns, not significant; *** $p < 0.001$, **** $p < 0.0001$; unpaired t-test).

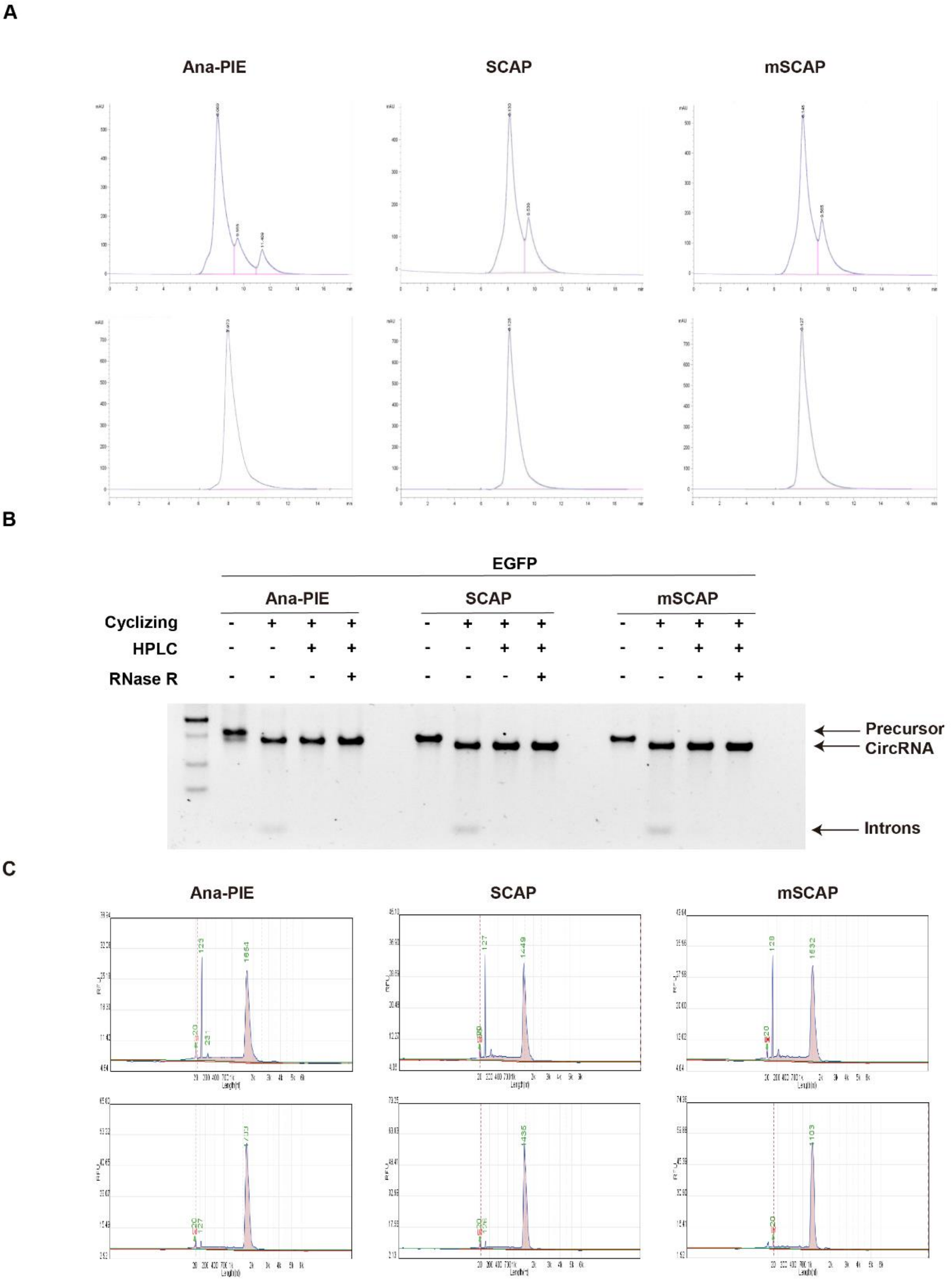

Figure S2. High-purity circRNAs.

(A) HPLC chromatograms of circEGFP before (top) and after (bottom) HPLC purification and RNase R digestion, confirming the high purity of circEGFP.

(B) Agarose gel electropherogram of circEGFP at different stages of purification, showing effective removal of linear precursors and introns.

(C) Capillary electrophoresis analysis of circEGFP before (top) and after (bottom) HPLC purification and RNase R digestion, confirming the purity and homogeneity of the final product.

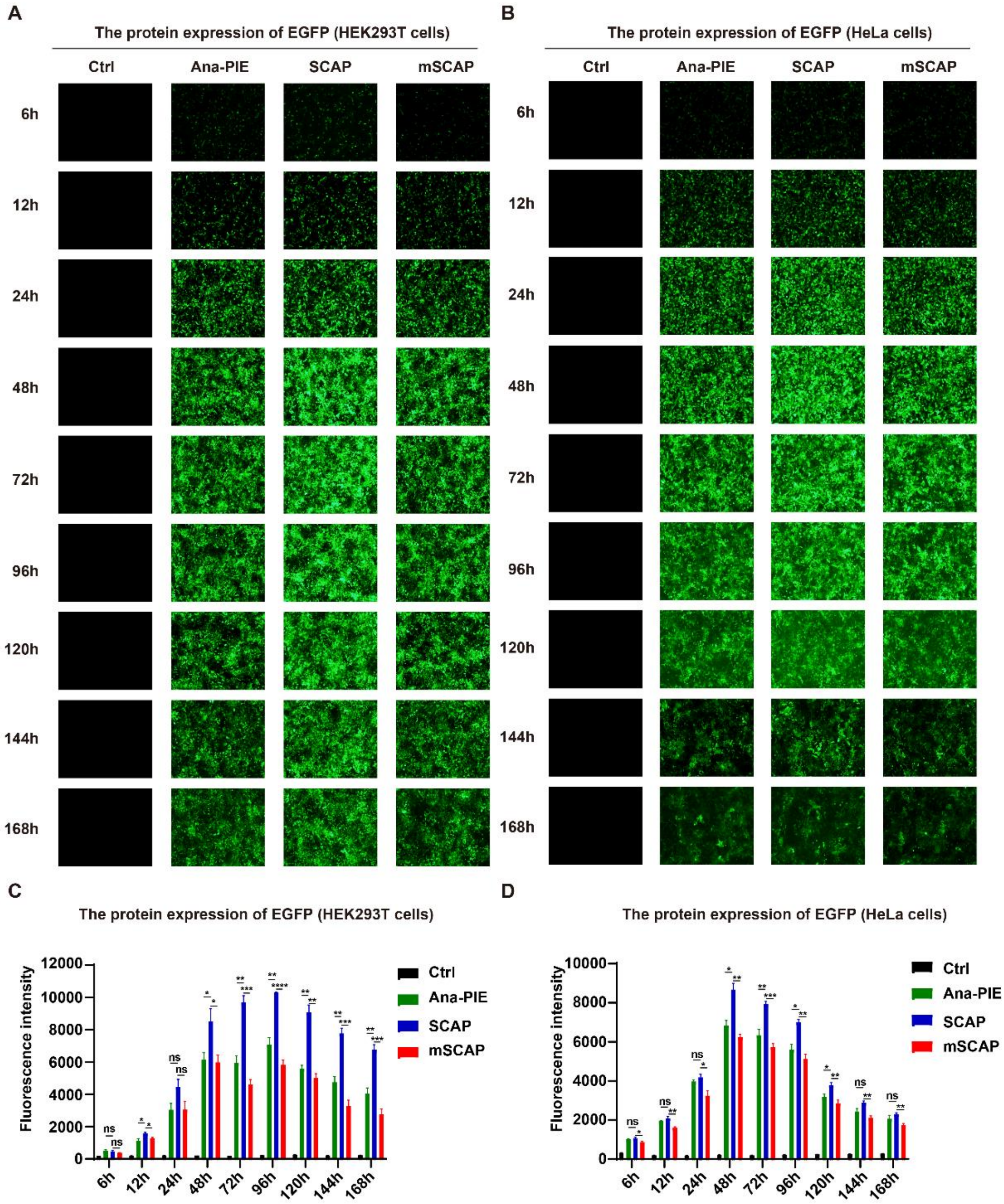

Figure S3. The protein production of EGFP in HEK293T and HeLa cells transfected with EGFP-encoding RNAs.

(A) Fluorescence microscopy images (GFP filter) of HEK293T cells from 6 hours to 168 hours after transfection with EGFP-encoding RNA.

(B) Fluorescence microscopy images (GFP filter) of HeLa cells from 6 hours to 168 hours after transfection with EGFP-encoding RNA.

(C) Fluorescence intensity quantification of HEK293T cells from 6 hours to 168 hours after transfection with EGFP-encoding RNA ($n = 3$; mean ± SEM).

(D) Fluorescence intensity quantification of HeLa cells from 6 hours to 168 hours after transfection with EGFP-encoding RNA ($n = 3$; mean ± SEM).

Note: ns, not significant, *$p<0.05$, **$p < 0.01$, ***$p < 0.001$, ****$p < 0.0001$; unpaired t-test.

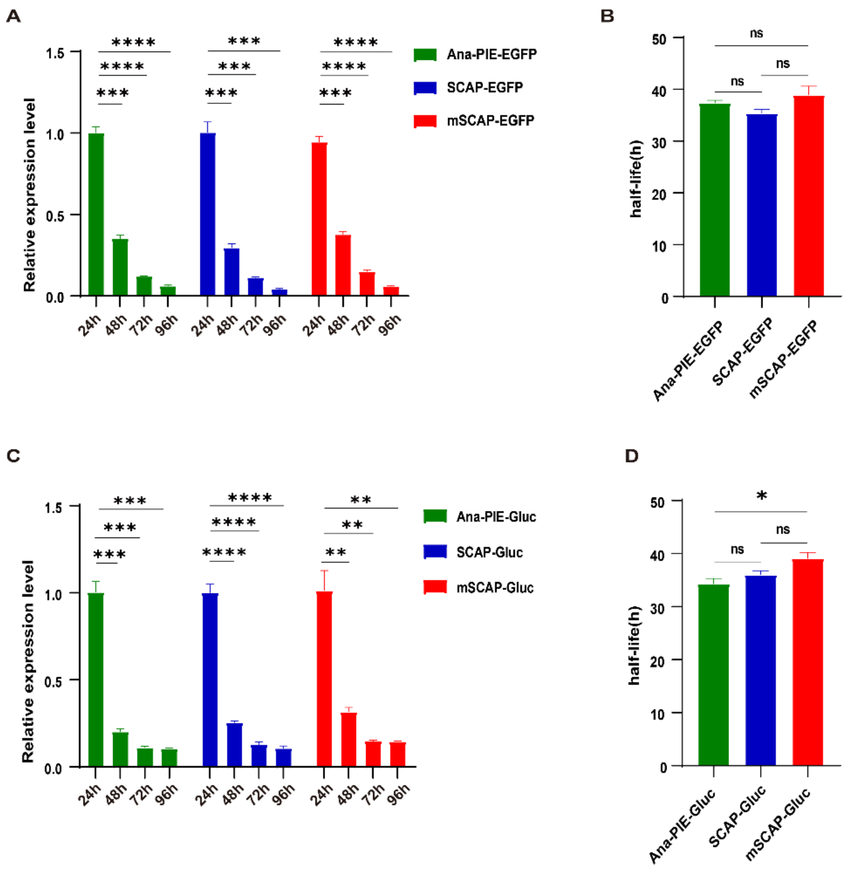

Figure S4. Stability testing of circRNAs in HeLa cells.

(A) The relative expression level of circEGFP, with data normalized to the expression level of the 24-hour time point. (n = 3; mean ± SEM)

(B) The half-life of circEGFP produced by the SCAP, mSCAP, and Ana-PIE systems (n = 3; mean ± SEM).

(C) The relative expression level of circGluc, with data normalized to the expression level of the 24-hour time point (n = 3; mean ± SEM)

(D) The half-life of circGluc produced by the SCAP, mSCAP, and Ana-PIE systems (n = 3; mean ± SEM).

Note: ns, not significant, *$p<0.05$, **$p < 0.01$, ***$p < 0.001$, ****$p < 0.0001$; unpaired t-test.

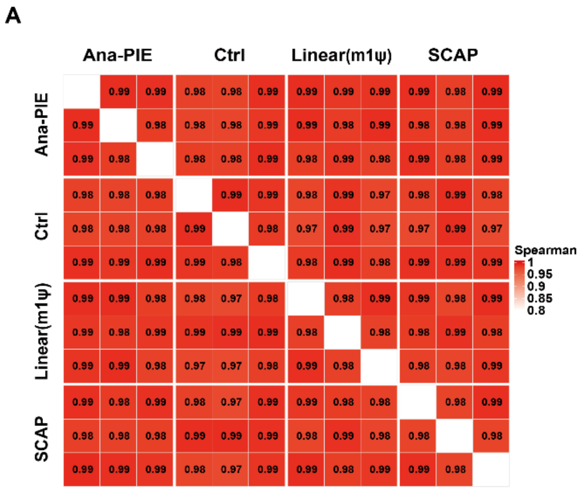

Figure S5. Spearman correlation coefficients for the Ctrl, Ana-PIE, Linear(m1ψ) and SCAP groups.

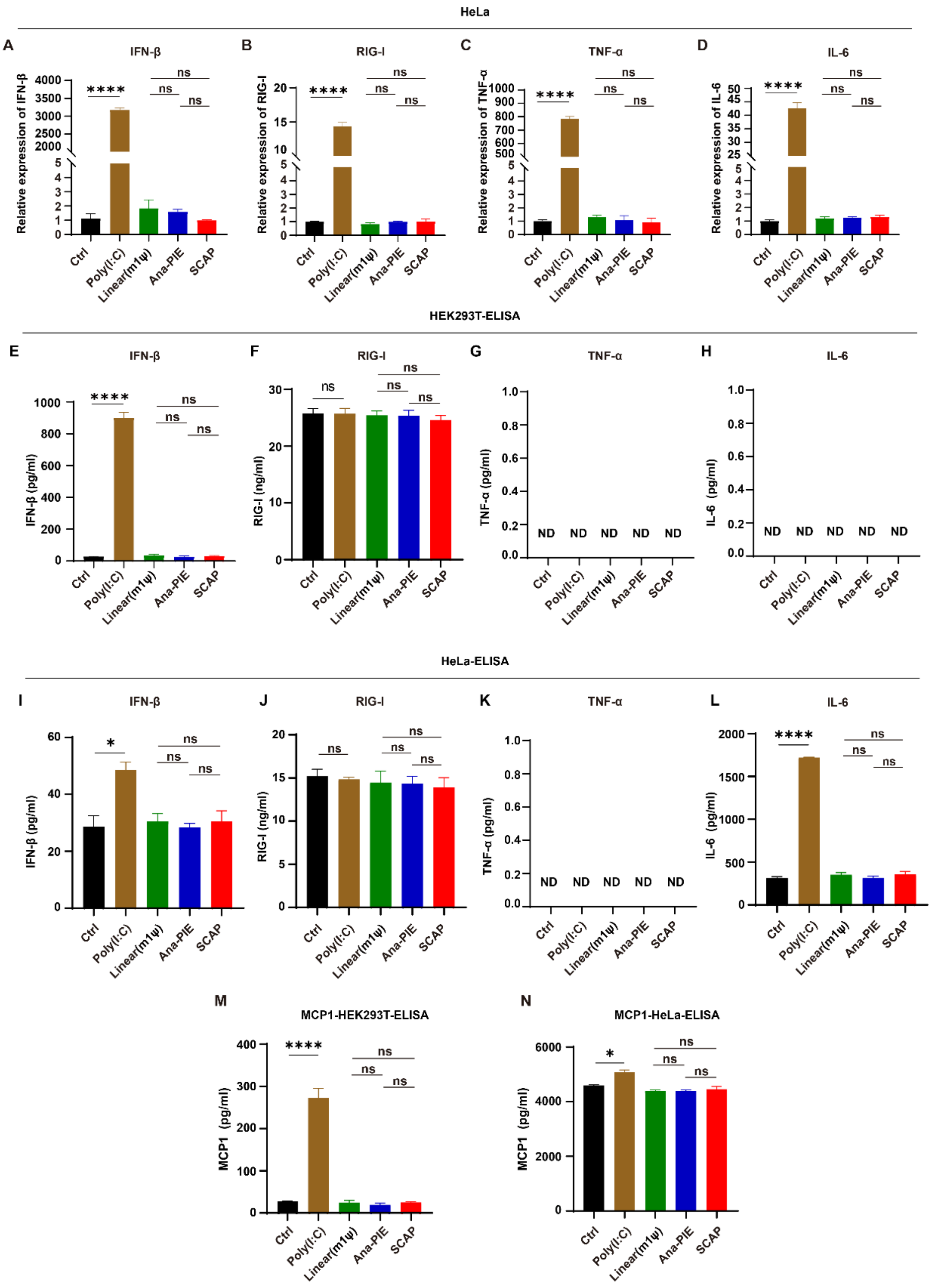

Figure S6. Detection of immune-related factors.

(A-D) mRNA expression of IFN-β, RIG-I, TNF-α, and IL-6 measured by qPCR transfected with Gluc-encoding RNAs at 24 hours in HeLa cells. (n = 3; mean ± SEM).

(E-H, M) Protein expression of IFN-β, RIG-I, TNF-α, IL-6 and MCP1 measured by ELISA transfected with Gluc-encoding RNAs at 48 hours in HEK293T cells ($n = 3$; mean ± SEM).

(I-L, N) Protein expression of IFN-β, RIG-I, TNF-α, IL-6 and MCP1 measured by ELISA transfected with Gluc-encoding RNAs at 48 hours in HeLa cells ($n = 3$; mean ± SEM).

Note: Poly(I:C) was served as a positive control. ns, not significant, * $p < 0.05$, **** $p < 0.0001$; unpaired t-test; ND, not detected.

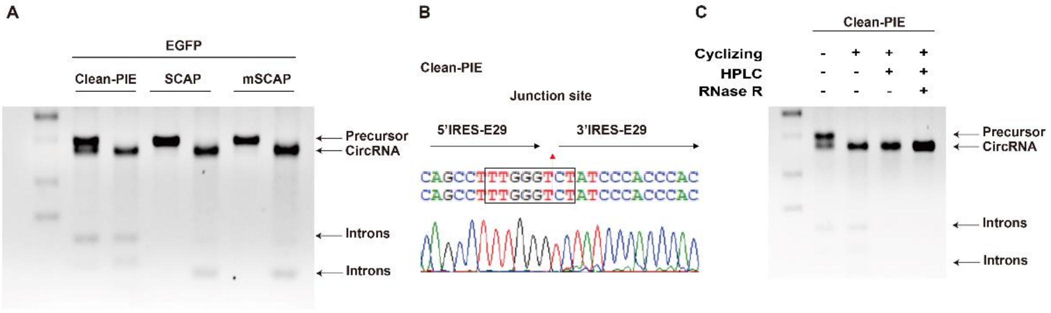

Figure S7. CircRNA synthesis via the Clean-PIE circularization system.

(A) Agarose gel electrophoresis of circEGFP produced by the Clean-PIE, SCAP, and mSCAP systems.

(B) Sanger sequencing of the circEGFP circularization junctions.

(C) Agarose gel electropherogram of circEGFP at different stages of purification, showing effective removal of linear precursors and introns.
